# Reprogramming the Immunosuppressive Microenvironment in Triple‐Negative Breast Cancer via FAP‐Targeted Nanoprobes for Multimodal Imaging and Photothermal Therapy

**DOI:** 10.1002/adma.74089

**Published:** 2026-07-15

**Authors:** Ling Zhan, Yanhong Chen, Jingjing Hu, Chunting Wang, Huanhuan Liu, Defan Yao, Dengbin Wang

**Affiliations:** ^1^ Department of Radiology, Xinhua Hospital Shanghai Jiao Tong University School of Medicine Shanghai China; ^2^ Faculty of Medical Imaging Technology College of Health Science and Technology Shanghai Jiao Tong University School of Medicine Shanghai China

**Keywords:** cancer‐associated fibroblasts, immunotherapy, photothermal therapy, triple‐negative breast cancer

## Abstract

Triple‐negative breast cancer (TNBC) poses a major clinical challenge because of its aggressive behavior, high likelihood of metastasis, and absence of specific therapeutic targets. The immunosuppressive tumor microenvironment, largely influenced by cancer‐associated fibroblasts (CAFs) through fibroblast activation protein (FAP)‐mediated extracellular matrix (ECM) remodeling and cytokine secretion, contributes to TNBC's resistance to immunotherapy. In response to this challenge, we have developed FAP‐targeted nanoprobes (FAP‐IR1061) that incorporate second near‐infrared (NIR‐II) fluorescence/magnetic resonance (MR)/photoacoustic (PA) imaging capabilities alongside highly efficient NIR‐II photothermal conversion for precision theranostics. This platform facilitates a tripartite therapeutic approach: (1) selective ablation of CAFs and dismantling of the ECM to overcome physical barriers; (2) spatiotemporal induction of immunogenic cell death through the release of damage‐associated molecular patterns; (3) functional repolarization of tumor‐associated neutrophils toward antitumor N1 phenotypes. When used in conjunction with anti‐programmed cell death protein 1 (αPD‐1) therapy, this strategy achieves over 80% suppression of both primary and distant tumors by enhancing the infiltration of cytotoxic T lymphocytes. Collectively, these effects promote the establishment of long‐term antitumor immune memory, which facilitates tumor eradication and prevents tumor recurrence.

## Graphical abstract

Targeting cancer‐associated fibroblasts (CAFs) with fibroblast activation protein (FAP)‐directed nanoprobes for multimodal imaging and photothermal remodeling of the immunosuppressive microenvironment to enhance immunotherapy in triple‐negative breast cancer (TNBC). We developed FAP‐targeted nanoprobes integrating second near‐infrared (NIR‐II) fluorescence/magnetic resonance (MR)/photoacoustic (PA) imaging and NIR‐II photothermal conversion for TNBC precision theranostics, addressing immunotherapy resistance caused by CAFs‐mediated immunosuppressive tumor microenvironment (TME). Its theranostic mechanism includes: (1) selective CAFs ablation and extracellular matrix (ECM) disruption; (2) induction of immunogenic cell death (ICD) to enhance tumor immunogenicity; (3) reprogramming tumor‐associated neutrophils (TANs) from N2 to N1 phenotypes. Combined with anti‐programmed cell death protein 1 (αPD‐1), this strategy achieves over 80% suppression of primary TNBC and distant metastases, and induces long‐term antitumor immune memory.

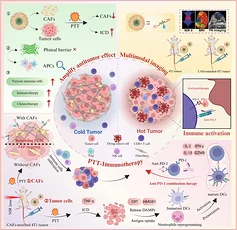

## 1 Introduction

Triple‐negative breast cancer (TNBC) represents a formidable clinical challenge, characterized by its aggressive nature, high metastatic potential, and lack of expression of estrogen receptor (ER), progesterone receptor (PR), and human epidermal growth factor receptor 2 (HER2) [1, 2]. This molecular profile renders TNBC unresponsive to established endocrine therapies and HER2‐targeted agents, leaving cytotoxic chemotherapy as the primary systemic treatment option. However, chemotherapy often yields suboptimal responses, is associated with significant toxicity, and a high proportion of TNBC patients develop resistance or experience rapid recurrence. The inherent heterogeneity and aggressive biology of TNBC contribute to a notoriously poor prognosis compared to other breast cancer subtypes, underscoring the urgent need for novel, targeted therapeutic strategies. The emergence of cancer immunotherapy, particularly immune checkpoint blockade (ICB) targeting pathways like programmed cell death protein 1 (PD‐1) / programmed death‐ligand 1 (PD‐L1), has revolutionized oncology [1, 3]. However, its clinical success in TNBC has been limited and inconsistent. A major factor contributing to this therapeutic resistance is the characteristic immunosuppressive tumor microenvironment (TME) prevalent in TNBC, often classified as “immunologically cold” [4, 5, 6]. These tumors exhibit poor infiltration of cytotoxic T lymphocytes (CTLs), impaired antigen presentation, and an abundance of immunosuppressive cell populations and factors [6, 7].

Cancer‐associated fibroblasts (CAFs) are pivotal components of this immunosuppressive TME [8, 9, 10]. They are not merely passive structural components but active players in tumor progression, metastasis, and the immunosuppressive TME by remodeling the extracellular matrix (ECM) and releasing various cytokine. Alpha‐smooth muscle actin (α‐SMA), fibronectin (FN), and Type I collagen protein, collectively participate in the construction of ECM in tumors, mainly form a physical barrier, constraining immune penetration and leading to various therapeutic resistances [11]. Furthermore, CAFs produce a plethora of immunosuppressive cytokines, chemokines, and growth factors (e.g., TGF‐β, Interleukin 6 (IL‐6), CXCL 12) that actively suppress antitumor immunity, promote angiogenesis, sustain cancer stem cells, and directly inhibit T cell function [10, 12, 13]. Critically, CAFs overexpress fibroblast activation protein (FAP), a type II transmembrane serine protease implicated in ECM degradation and tumor progression, rendering it a promising therapeutic target [14]. FAP exhibits tumor‐restricted expression, with selective upregulation in stromal fibroblasts of solid tumors (e.g., breast cancer, pancreatic ductal adenocarcinoma, gastric cancer), while remaining virtually undetectable in normal tissues and benign lesions [9, 12, 15, 16]. This specificity positions FAP as both a diagnostic biomarker for CAFs identification and a therapeutic vulnerability [14, 17, 18, 19, 20]. Despite their central role in TME dysregulation, effective strategies to target CAFs and convert immunologically “cold” tumors to “hot” remain an unmet clinical need.

Photothermal therapy (PTT), utilizing light‐absorbing agents to generate localized heat when exposed to second near‐infrared (NIR‐II) laser irradiation, making it a promising noninvasive or minimally invasive modality for solid tumor ablation [21, 22, 23, 24]. The hyperthermia induced by PTT can directly kill tumor cells and, importantly, has the potential to induce immunogenic cell death (ICD). ICD is characterized by the release of damage‐associated molecular patterns (DAMPs) such as calreticulin (CRT) and high mobility group box 1 (HMGB 1), which can stimulate dendritic cell (DC) maturation, enhance antigen presentation, and ultimately prime antitumor T cell responses [25]. However, the efficacy of conventional PTT is often hampered by several limitations: (1) poor penetration of light, especially in the traditional NIR‐I window (700–900 nm), limiting its effectiveness in deep‐seated tumors; (2) suboptimal accumulation and retention of photothermal agents within the tumor tissue; (3) the development of thermotolerance and heat shock responses in surviving tumor cells; (4) the protective and immunosuppressive role of the CAFs‐rich stroma and dense ECM, which physically shields tumor cells and impedes immune cell access; and (5) the potential for incomplete ablation leading to recurrence and metastasis.

Combining PTT with immunotherapy, particularly ICB, holds great promise to overcome these limitations by leveraging the localized tumor destruction, TME remodeling and enhanced systemic immune activation, which can then be amplified and sustained by ICB. However, effectively integrating these modalities requires overcoming the barriers posed by the TME, especially the CAFs‐dominated stroma. Thus, the development of advanced theranostic platforms is crucial for advancing precision oncology. Multimodal imaging allows for comprehensive tumor characterization, precise localization, treatment guidance, and real‐time monitoring of therapeutic response [26, 27]. For PTT‐immunotherapy combinations, imaging is indispensable for ensuring accurate laser targeting, assessing photothermal agent biodistribution, visualizing the extent of ablation, and tracking immune dynamics [28]. Near‐infrared‐II (NIR‐II, 900–1700 nm) PTT and fluorescence imaging offer significant benefits over the NIR‐I window, including superior penetration depth, higher spatial resolution, and more precise theranostic capabilities for deep tumors compared to NIR‐I [29]. Photoacoustic (PA) imaging provides high‐resolution images of optical absorption contrast at greater depths that surpass those of purely optical techniques, offering valuable functional and structural information [30, 31]. Magnetic resonance (MR) imaging delivers unparalleled soft‐tissue contrast and anatomical delineation [32, 33]. Integrating these complementary imaging modalities into a unified platform provides a powerful tool for guiding complex combination therapies like PTT‐immunotherapy. Nevertheless, designing biocompatible, target‐specific multifunctional probes exhibiting high photothermal conversion efficiency within the optimal NIR window remains a substantial engineering challenge.

**SCHEME 1:**
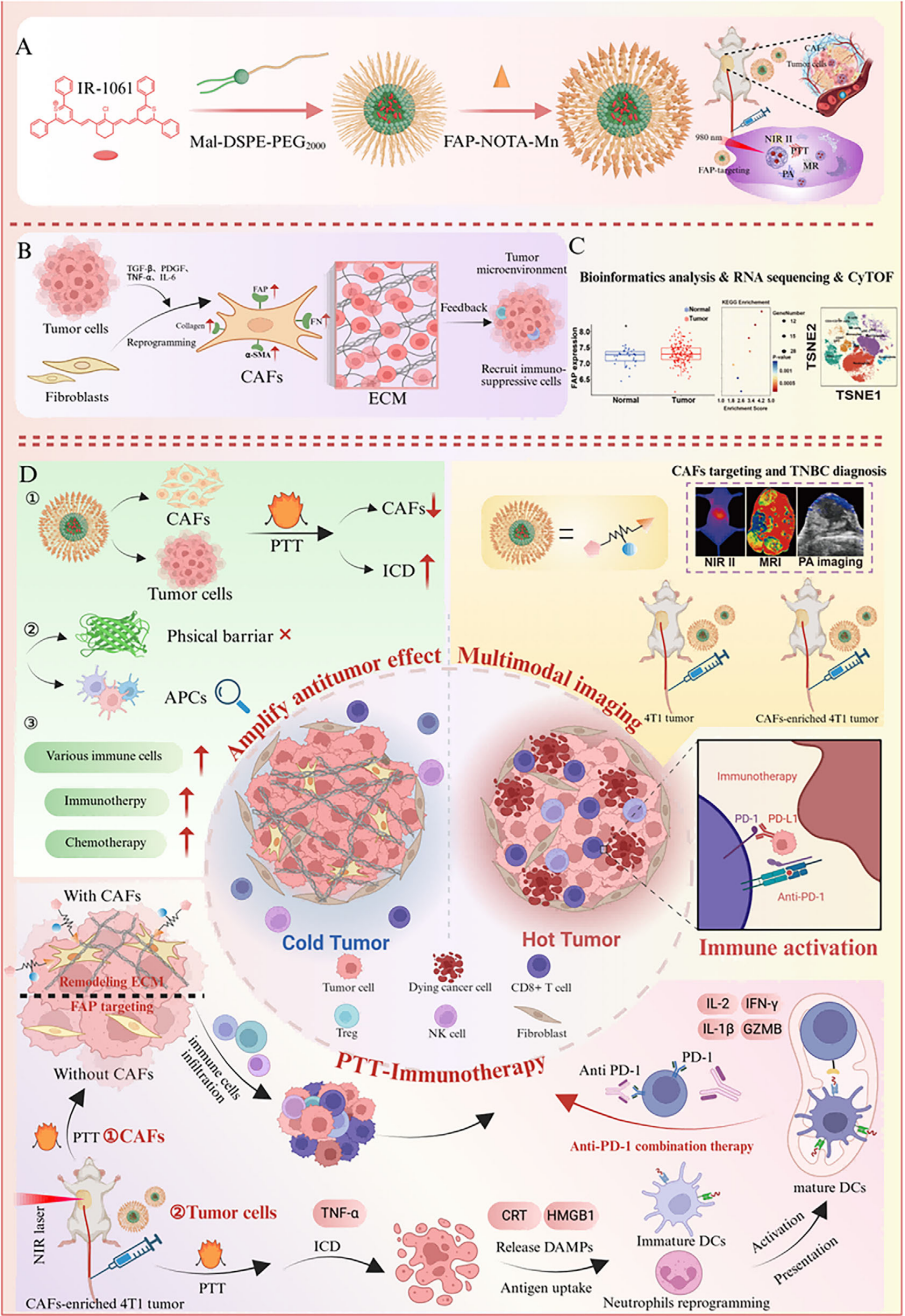
Illustration of FAP‐targeted imaging and PTT‐immunotherapy of TNBC. (A) Chemical structure of FAP‐targeted probes FAP‐IR1061. (B) CAFs are activated by TGF‐β and kinds of cytokines, leading to ECM remodeling and recruitment of immunosuppressive cells, forming a feedback loop that facilitates tumor progression. (C) Bioinformatics analysis, RNA sequencing, CyTOF, and other techniques are used to assist in investigating the role of CAFs in the TME of TNBC, as well as the changes in the TME after PTT‐immunotherapy. (D) In vivo NIRF II/MR/PA imaging was used to achieve comprehensive and multidimensional visual evaluation of FAP‐IR1061 distribution, and TME in living organisms after tail vein injection. Laser irradiation activates PTT, remodeling the ECM and improving the immunosuppressive TME. PTT also promotes tumor cell ICD, antigen recognition, and presentation, leading to DC activation, neutrophil reprogramming, and T cell activation. Administering αPD‐1 further boosts T cells' ability to kill and clear tumor cells.

In this study, we introduce the rational design and synthesis of multifunctional FAP‐IR1061 nanoprobes (NPs) that innovatively integrate precision CAFs ablation with triple‐modal imaging guidance to enhance synergistic PTT‐immunotherapy. This platform was meticulously engineered by conjugating a high‐affinity FAP‐targeting ligand to a DSPE‐IR1061 complex, leveraging the tumor‐selective overexpression of FAP on CAFs. The system integrates NIR‐II fluorescence, PA, and MR imaging modalities to facilitate real‐time and high‐resolution tumor delineation. Notably, the IR1061 chromophore acts as a highly efficient NIR‐II photothermal converter, imparting the NPs with exceptional photothermal conversion efficiency and deep‐tissue penetration capabilities for effective PTT (Scheme 1). In addition to directly targeting CAFs, these NPs actively disrupt the tumor ECM barrier, thereby enhancing immune cell infiltration [13, 34, 35]. Simultaneously, PTT induces robust ICD through spatiotemporal release of DAMPs that catalyze dendritic cell maturation, amplify antigen presentation, and activate adaptive immunity [36, 37]. Notably, we discovered a concomitant phenotype switch of tumor‐associated neutrophils (TANs) from protumorigenic (N2) to antitumorigenic (N1) states, which is a previously uncharacterized mechanism critical for immune activation [38, 39]. Synergistically, anti‐PD‐1 (αPD‐1) coadministration augments T cell‐mediated tumor clearance, establishing a self‐reinforcing PTT‐immunotherapy cycle [40]. This CAFs‐targeted strategy further confer multimodal therapeutic advantages: suppressing drug‐resistance pathways [41], normalizing pathological vasculature [42], attenuating cellular stress [41], and inhibiting metastatic dissemination. Consequently, CAFs‐targeting PTT‐immunotherapy demonstrated efficacy in inhibiting primary tumor growth, converting immunologically ‘cold’ tumors into ‘hot’ lesions, characterized by the infiltration of mature dendritic cells and CTLs, ultimately resulting in the restoration of immune tolerance in TNBC. This research provides a novel technical approach and theoretical basis for the precise diagnosis, effective treatment, and enhancement of immunotherapy efficacy in TNBC.

## 2 Results

### 2.1 Analysis of FAP Expression in TNBC and Its Impact on Survival

**FIGURE 1:**
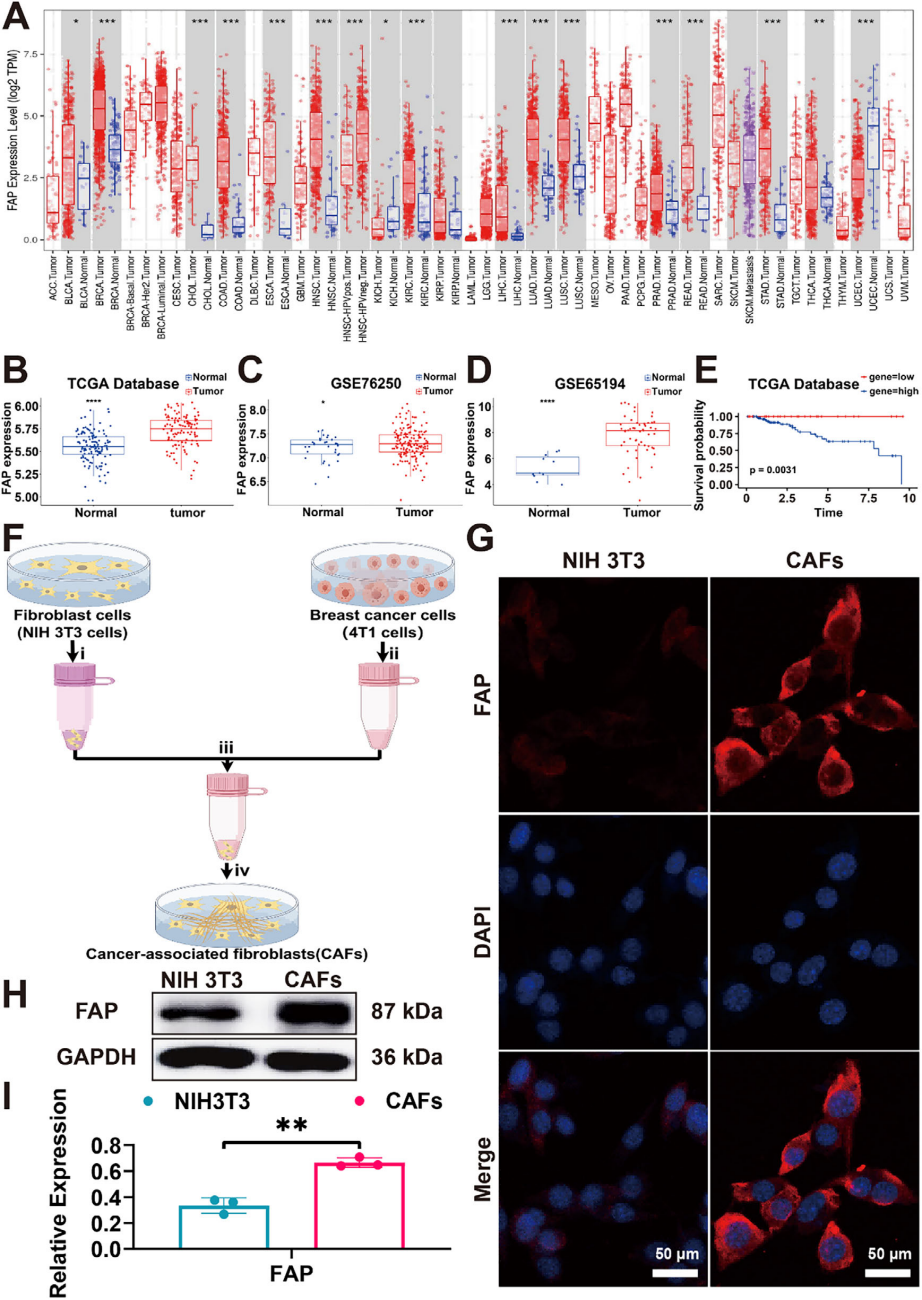
Bioinformatics analysis of breast cancer and experimental validation of CAFs. (A) FAP expression levels across various cancer types and breast cancer. (B–D) FAP expression in tumor versus para‐cancerous tissues of TNBC patients in the Cancer Genome Atlas (TCGA) databases and Gene Expression Omnibus (GEO) databases. (E) The correlation between the expression levels of FAP and prognosis among TNBC patients in the TCGA database. (F) A schematic illustration of the construction of the CAFs model. (G) Immunofluorescence images of FAP in NIH 3T3 cells and CAFs, FAP: red channel, *λ*_ex_ = 561 nm, *λ*_em_ = 600–650 nm; DAPI: blue channel, *λ*_ex_ = 405 nm, *λ*_em_ = 450–500 nm. Scale bar: 50 µm. (H) Western blot images of FAP in NIH 3T3 cells and CAFs. (I) Quantitative analysis of FAP protein expression from Western blot, *n* = 3, data present as mean ± standard deviation (SD) (**p < 0.01).

The FAP expression levels of pan‐cancer and breast cancer were analyzed by bioinformatics, which revealed a marked elevation in breast cancer patients compared to normal subjects, with statistically significant results (Figure 1A). Intriguingly, data from the Cancer Genome Atlas (TCGA) databases and Gene Expression Omnibus (GEO) databases (GSE76250, GSE65194) demonstrated significantly higher FAP expression in tumor tissues of TNBC (Figure 1B–D). What's more, data from TCGA databases revealed a significantly positive correlation between the high expression levels of FAP and poor prognosis among TNBC patients (Figure 1E). Upon performing cellular fractionation and FAP visualization from single‐cell data from GEO CAFs (GSE199515), it was seen that FAP was predominantly expressed on fibroblast subpopulations (Figure S1). Previous literature has indicated that tumor‐secreted TGF‐β is a major catalyst for fibroblast activation [43]. Culturing NIH 3T3 cells with conditioned medium (CM) from mouse breast cancer cells‐induced their transformation into CAFs (Figure 1F). In vitro fluorescence experiments CAFs abundantly express FAP compared to NIH 3T3 cells (Figure 1G). Western blot analysis also showed increased expression of FAP in CAFs (Figure 1H,I).

### 2.2 Activated CAFs Contribute to Excessive ECM Production and Tumor Mechanical Stress Formation

**FIGURE 2:**
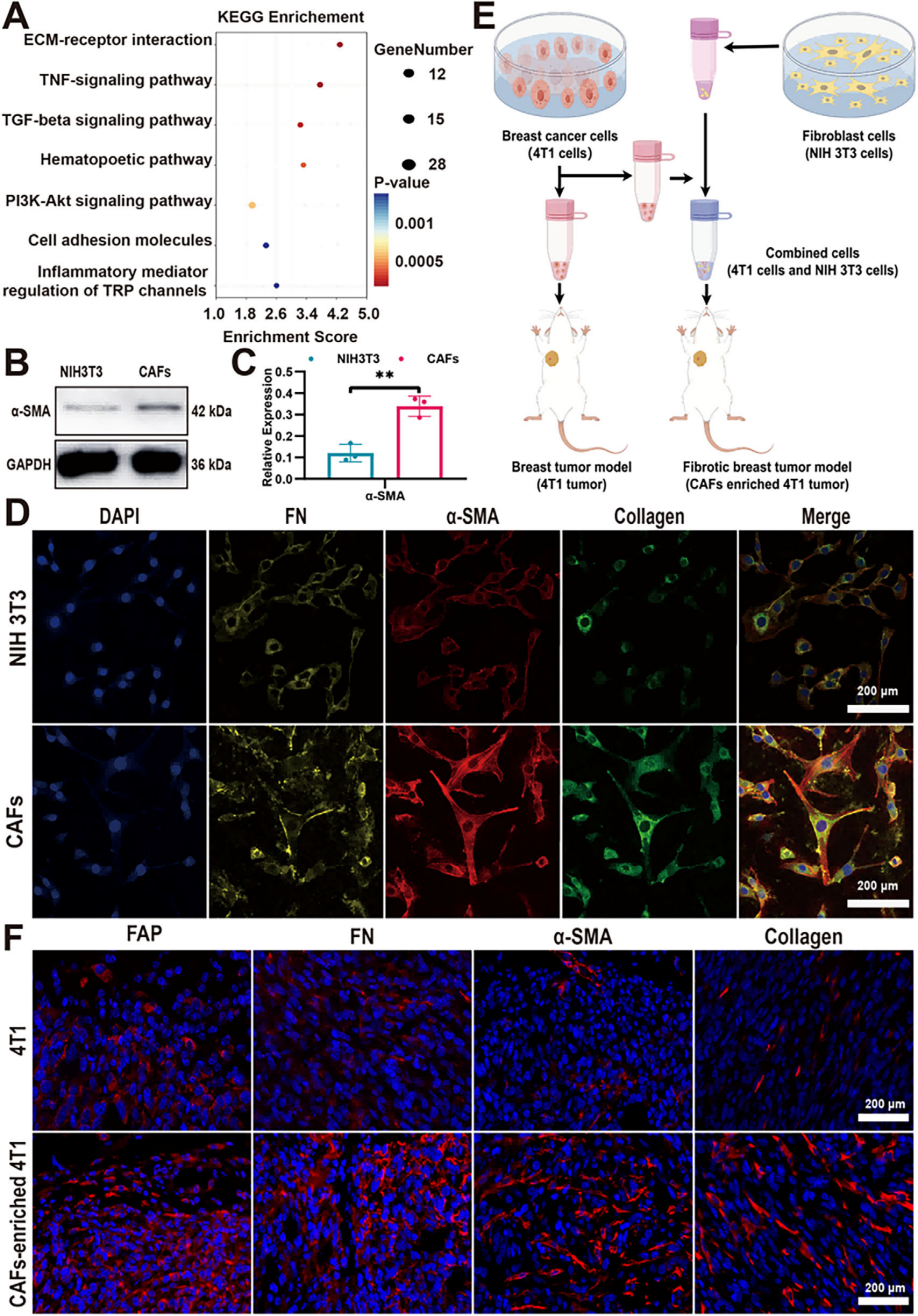
The experimental validation of the tumor extracellular matrix. (A) Kyoto Encyclopedia of Genes and Genomes (KEGG) pathway analyses of differential genes in NIH 3T3 and CAFs. (B,C) The expression levels of α‐SMA protein in NIH 3T3 cells and CAFs, *n* = 3, data present as mean ± SD (**p < 0.01). (D) mIF images of α‐SMA, FN, and collagen in NIH 3T3 cells and CAFs, α‐SMA: red channel, *λ*_ex_ = 640 nm, *λ*_em_ = 655–750 nm; FN: yellow channel, *λ*_ex_ = 561 nm, *λ*_em_ = 570–620 nm; and collagen: green channel, *λ*_ex_ = 488 nm, *λ*_em_ = 500–550 nm; DAPI: blue channel, *λ*_ex_ = 405 nm, *λ*_em_ = 450–500 nm. Scale bar: 200 µm. (E) A schematic illustration of the construction of a CAFs‐enriched 4T1 breast cancer model and a standard 4T1 solid tumor model. (F) Immunofluorescence imaging of FAP, α‐SMA, FN, and collagen in tumor tissues of mice with CAFs‐enriched 4T1 tumors and control tumors, FAP, α‐SMA, FN, and collagen: red channel, *λ*_ex_ = 561 nm, *λ*_em_ = 600–650 nm; DAPI: blue channel, *λ*_ex_ = 405 nm, *λ*_em_ = 450–500 nm. Scale bar: 200 µm.

Kyoto Encyclopedia of Genes and Genomes (KEGG) pathway analyses and the Gene Ontology (GO) functional analysis histogram revealed that when CAFs were activated, the differential genes associated with FAP were mainly enriched in pathways related to epithelial–mesenchymal transition (EMT), such as the ECM pathway [10] (Figure 2A and Figure S2). Western blots experiments demonstrated that CAFs exhibited a significantly higher expression of α‐SMA compared to NIH 3T3 cells (Figure 2B,C). Cellular multiplex immunofluorescence (mIF) assays showed that there was a higher expression of α‐SMA, FN, and collagen on the surface of CAFs (Figure 2D). To simulate the clinical scenario of CAF enrichment in the TME of breast cancer, 4T1 cells and NIH 3T3 cells were cocultured and then implanted into the right second mammary fat pads of BALB/c mice, thereby establishing a CAFs‐enriched 4T1 breast cancer model. Meanwhile, a standard 4T1 solid tumor model was constructed as a control (Figure 2E). In vivo fluorescence imaging experiments further confirmed an increased expression of FAP, α‐SMA, FN, and collagen in the tumor tissues of mice with CAFs‐enriched tumors (Figure 2F). These findings suggest that activated CAFs significantly upregulate the expression of ECM proteins and increased tumor mechanical stress, resulting in limited drug penetration and immune cell infiltration.

### 2.3 Preparation and Characterization of the FAP‐IR1061 Nanoparticles

**FIGURE 3:**
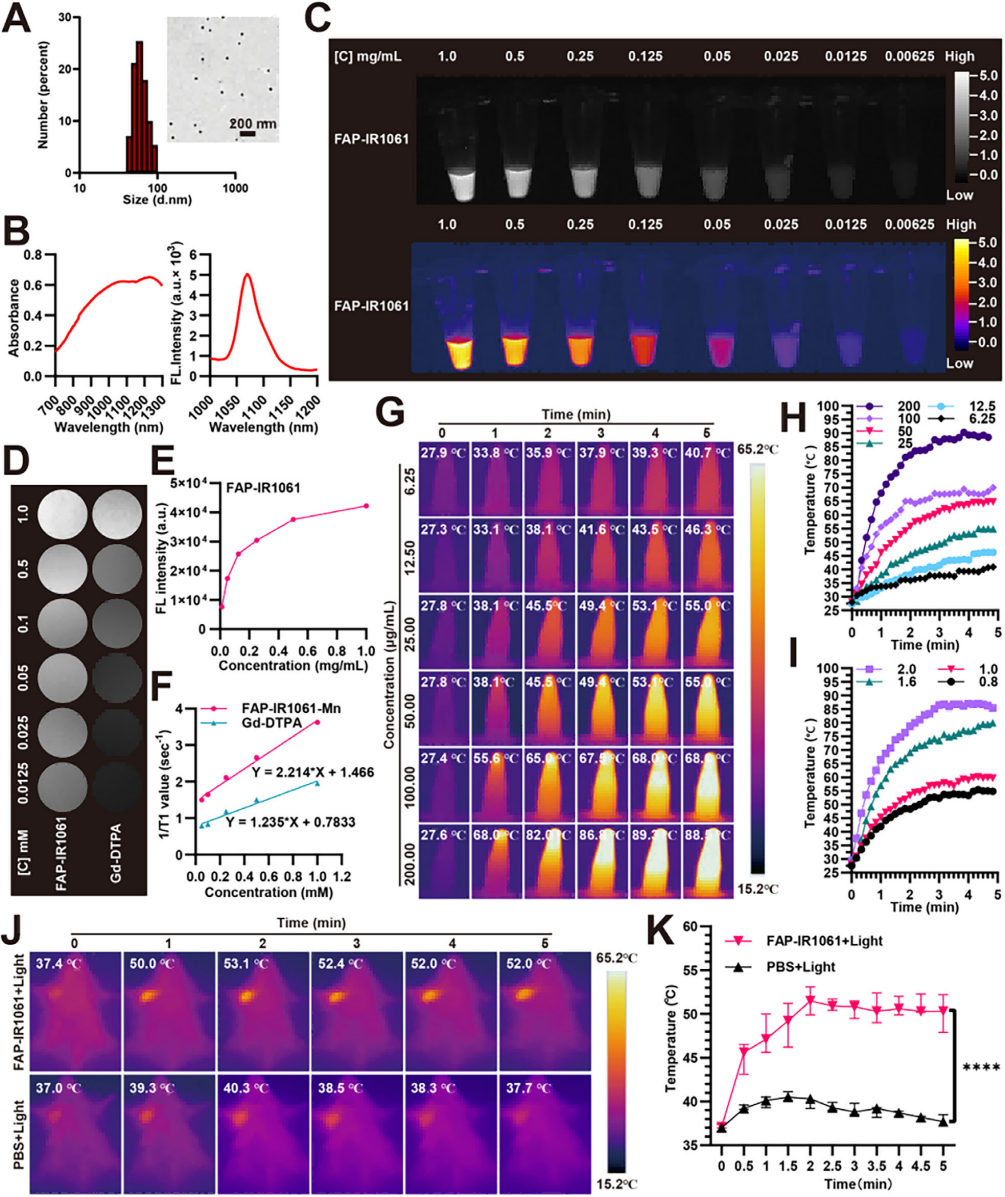
Preparation and characterization of FAP‐IR1061 NPs. (A) Representative TEM images (scale bar: 200 µm) and DLS profiles of FAP‐IR1061 NPs. (B) Absorption (*λ*_ab_: 700–1300 nm), and fluorescence spectra (*λ*_em_: 1000–1200 nm, *λ*_ex_: 980 nm) of FAP‐IR1061 NPs in PBS. (C) NIR‐II fluorescence images and pseudocolor images of the FAP‐IR1061 NPs (0.00625−1.0 mg mL^−1^) in PBS solution under 980 nm laser illumination (1.0 W cm^−2^). (D) The MR images of FAP‐IR1061 and Gd‐DTPA at different concentrations (0.0125−1.0 mm). (E) Quantitative curve of fluorescence signals for FAP‐IR1061 NPs (0.00625−1.0 mg mL^−1^) in PBS solution under 980 nm illumination. (F) Plot of longitudinal relaxation rate (1/T1) versus concentrations (0.0125−1.0 mm). (G) The temperature image of the FAP‐IR1061 NPs solutions at different concentrations (6.25–200 µg mL^−1^) under 980 nm laser irradiation (1.0 W cm^−^^2^). (H,I) Temperature change curves of the FAP‐IR1061 NPs solution under 980 nm laser irradiation at different concentration (6.25–200 µg mL^−1^, 1.0 W cm^−^^2^) (H) and laser power densities (0.8–2.0 W cm^−^^2^, 50 µg mL^−1^) (I). (J,K) Infrared thermal images (J) and temperature change curves (K) of CAFs‐enriched 4T1 tumor‐bearing mice at 6 h postinjection of FAP‐IR1061 NPs (1.0 mg mL^−1^, 100 µL) or PBS (100 µL), following 980 nm laser irradiation (1.0 W cm^−^^2^) for 5 min (**** *p* ＜ 0.0001).

We first synthesized the FAP‐targeted peptide FAP‐1,4,7‐triazacyclononane‐1,4,7‐triacetic acid (NOTA)‐Mn. Subsequently, FAP‐NOTA‐Mn and the NIR‐II emissive dye IR1061 were co‐encapsulated into 1,2‐distearoyl‐sn‐glycero‐3‐phosphoethanolamine‐N‐[maleimide(polyethylene glycol)‐2000] (Mal‐DSPE‐PEG_2000_) nanoparticles to form FAP‐IR1061 NPs [44]. Following a similar strategy, IR1061 encapsulated in Mal‐DSPE‐PEG_2000_ alone served as a nontargeted control. As shown in Figure 3A, the FAP‐IR1061 NPs exhibited a typical spherical morphology in transmission electron microscopy (TEM) images and a monodispersed size distribution according to dynamic light scattering (DLS) analysis [45]. Specifically, the FAP‐IR1061 NPs had a size of approximately 38 nm under TEM and 60 nm as measured by DLS. Similarly, the corresponding sizes of IR1061 NPs were 30 and 52 nm, respectively (Figure s3 A). As shown in Figure S3B,C, the DLS results demonstrate that the particle sizes of both FAP‐IR1061 NPs and IR1061 NPs remained stable throughout the 7 day incubation period, with no significant changes observed in phosphate‐buffered saline (PBS), RPMI‐1640 CM and 0.9% saline. Additionally, ultraviolet and visible (UV–Vis) spectrometer confirmed the successful encapsulation of FAP‐IR1061 molecules and IR1061 NPs, as the UV–Vis absorbance spectrum of FAP‐IR1061 NPs and IR1061 NPs showed their characteristic absorbance, which exhibited a broad NIR absorbance at 700–1300 nm (Figure 3B and Figure S3D) [46, 47]. Notably, this broad absorption profile encompasses the 980 nm excitation wavelength despite the loss of the sharp molecular peak characteristic. Both the FAP‐IR1061 NPs and IR‐1061 NPs emitted bright NIR‐II fluorescence, with emission peaks centered around 1061 nm under excitation by a 980 nm laser [48, 49] (Figure 3B and Figure S3E). Furthermore, zeta potential measurements shows that both FAP‐IR1061 and IR1061 were negatively charged (−0.06 ± 0.04; −0.46 ± 0.24 mV; respectively) attributed to the PEG coating (Figure S3F).

The NIR‐II images of FAP‐IR1061 NPs and IR‐1061 NPs (0.00625−1.0 mg mL^−1^) were captured using a NIR‐II fluorescence imaging system under 980 nm illumination (1.0 W cm^−2^) (Figure 3C and Figure S3G). Subsequently, samples of FAP‐IR1061 NPs and Gadolinium (Gd‐DTPA) were diluted in PBS to concentrations of 0.0125, 0.025, 0.05, 0.1, 0.5, and 1.0 mm for ex vivo T1‐weighted relaxometry measurements, which were conducted with a 9.4 T Bruker BioSpec (Figure 3D and Figure S3H). Fluorescence signals of FAP‐IR1061 NPs demonstrated a strong linear correlation with concentrations (Figure 3E and Figure S3I). The longitudinal relaxation rate (1/T1) was plotted against the concentrations (Figure 3F). Additionally, FAP‐IR1061 NPs exhibited potential for PA imaging under 980 nm. To evaluate this PA imaging performance, the PA intensity and the corresponding images of FAP‐IR1061 NPs were also investigated at different concentrations. As depicted in the Figure S3J–L, the PA signals from FAP‐IR1061 NPs showed a linear positive correlation with their concentrations, which ranged from 0.05–1.0 mg mL^−1^ (at 980 nm laser, 1.0 W cm^−2^).

FAP‐IR 1061 NPs are a type of photothermal agent, which absorbs specific wavelengths of light, enabling light penetration through tissues of approximately 5–10 mm [46, 47, 50, 51]. It converts light energy into thermal energy, triggering a mild increase in local temperature to achieve the effect of killing tumor cells. The photothermal conversion characteristics of FAP‐IR 1061 NPs were systematically evaluated, focusing on their notable heat generation capabilities under NIR‐II irradiation in vitro (Figure 3G). First, we evaluated the photothermal conversion abilities of FAP‐IR 1061 NPs (concentration range: 6.25–200 µg mL^−1^) by monitoring the temperature rise in the aqueous solutions using an infrared thermal camera, following exposure to a 980 nm laser (1.0 W cm^−^^2^). When the concentration was between 50 and 100 µg mL^−1^, the temperature can reach 60°C (Figure 3H). In contrast, the temperature increase of indocyanine green (ICG) at a concentration of 50 µg mL^−1^ (980 nm, 1.0 W cm^−2^) was less stable (Figure S4A). Then, the effect of different laser power densities (980 nm, range: 0.8–2.0 W cm^−2^) on the heating efficiency of the FAP‐IR 1061NPs (50 µg mL^−1^) was further analyzed. When the laser irradiance was 1.0 W cm^−^^2^, the temperature in vitro could reach 60°C (Figure 3I). These in vitro results indicated that a clear dependence on both the concentration and laser power density. Additionally, the temperature could then be rapidly reduced to the initial level when the nanoprobes was diluted to 50 µg mL^−1^ and 980 nm laser irradiation (1.0 W cm^−^^2^) was stopped after 5 minutes (min) of exposure (Figure S4B). The photothermal conversion efficiency (PCE) of FAP‐IR1061 NPs was calculated to be 64.2%. Moreover, after three consecutive heating–cooling cycles (each cycle: 980 nm laser irradiation at 1.0 W cm^−^^2^ for 5 min), the FAP‐IR 1061 NPs solution (50 µg mL^−1^) exhibited similar temperature rise and fall patterns, highlighting its excellent photothermal stability (Figure S4C).

Next, the photothermal conversion abilities of FAP‐IR1061 NPs into *vivo* were observed (Figure 3J). We intravenously injected PBS (100 µL) and FAP‐IR1061 NPs (1.0 mg mL^−1^, 100 µL) into mice via the tail vein and the tumor tissues were irradiated with 980 nm laser (1.0 W cm^−2^) for 5 min at 6 hour (h) postinjection. Then the temperature changes in the tumor area were documented through an infrared thermal camera (Figure 3J). The temperature at the tumor site in mice treated with FAP‐IR1061 NPs increased gradually and eventually stabilized at approximately 53°C which is sufficient to ablate tumor cells. In contrast, mice treated with PBS showed only a slight increase in temperature (Figure 3K). These results demonstrate that FAP‐IR1061 NPs can served as potential photothermal agents for tumor therapy.

### 2.4 In Vitro Cellular Uptake and Cytotoxicity Evaluation

**FIGURE 4:**
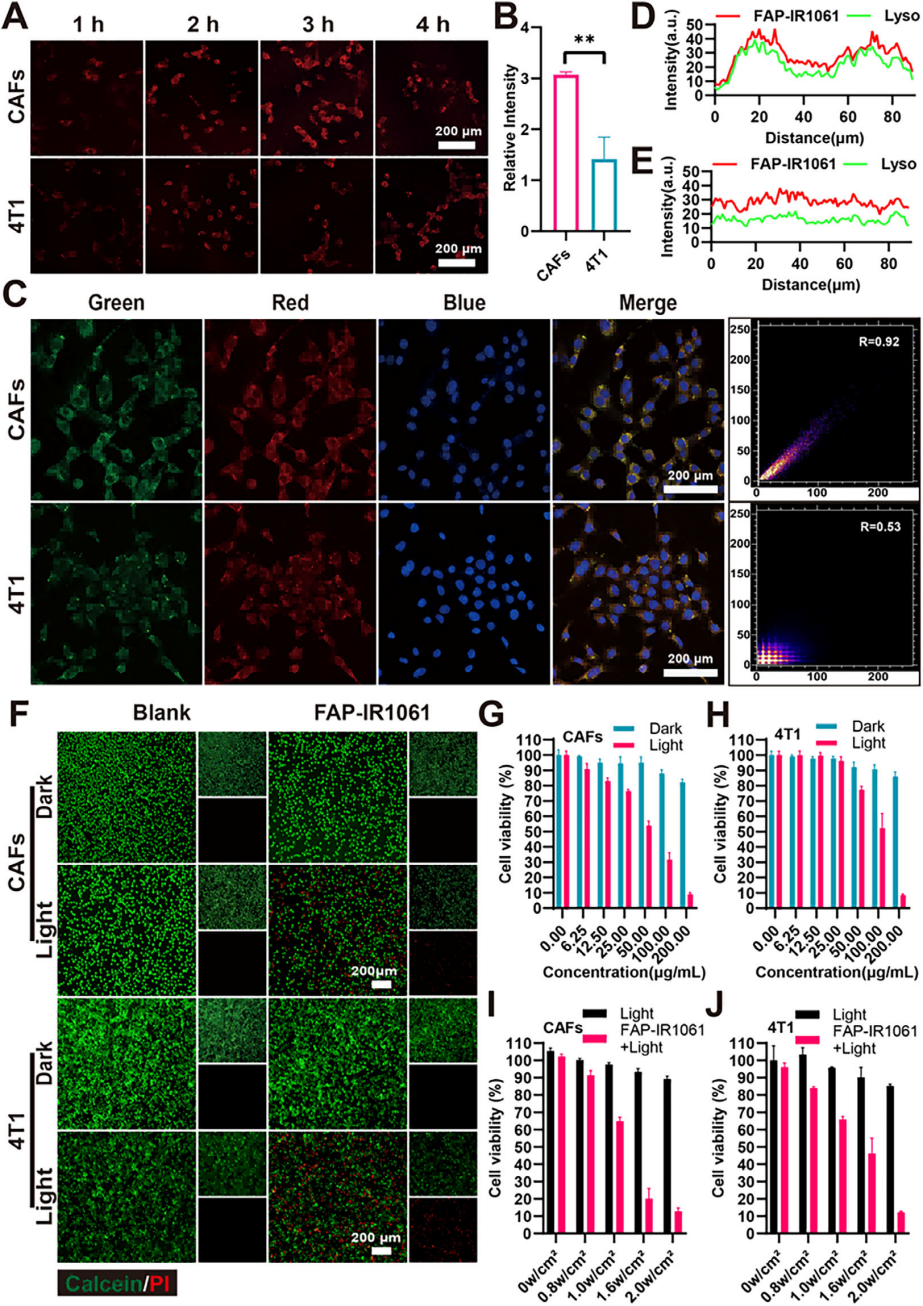
(A) **C**onfocal laser scanning microscope (CLSM) images of FAP‐IR1061 (Cy5 labeled) NPs (5.0 µm) in CAFs and 4T1 cells. FAP‐IR1061 (Cy5 labeled) NPs fluorescence is in the red channel (*λ*_ex_ = 640 nm, *λ*_em_ = 655–750 nm), and DAPI fluorescence is in the blue channel (*λ*_ex_ = 405 nm, *λ*_em_ = 450–500 nm). (B) Relative Fluorescence intensities of FAP‐IR1061 (Cy5 labeled) NPs in CAFs and 4T1 cells. Scale bar: 200 µm. *n* = 3, data present as mean ± SD (**p < 0.01). (C) CLSM images of CAFs and 4T1 cells treated with 5.0 µm FAP‐IR1061 (Cy5 labeled) NPs, co‐stained with 50 nm Lyso‐Tracker green. FAP‐IR1061 (Cy5 labeled) NPs fluorescence is in the red channel (*λ*_ex_ = 640 nm, *λ*_em_ = 655–750 nm), Lyso‐Tracker fluorescence is in the green channel (*λ*_ex_ = 488 nm, *λ*_em_ = 500−550 nm) and DAPI fluorescence is in the blue channel (*λ*_ex_ = 405 nm, *λ*_em_ = 450–500 nm). Scale bars = 200 µm. (D,E) Co‐localization curves of FAP‐IR1061 (Cy5 labeled) NPs and Lyso‐Tracker Green of (D) CAFs and (E) 4T1 cells. (F) Live−dead staining images of CAFs and 4T1 cells after treatment with FAP‐IR1061 NPs (100 µg mL^−1^) under dark or light condition (980 nm, 1.0 W cm^−2^, 3 min), calcein‐AM is in the green channel (*λ*_ex_ = 359 nm, *λ*_em_ = 517 nm), PI is in the red channel (*λ*_ex_ = 535 nm, *λ*_em_ = 617 nm). Scale bar: 200 µm. (G,H) Dose‐associated toxicity of (G) CAFs and (H) 4T1 cells treated with FAP‐IR1061 NPs (0–200 µg mL^−1^) under dark or light condition (980 nm, 1.0 W cm^−2^, 3 min). (I,J) Cell viabilities of (I) CAFs and (J) 4T1 cells treated with Light alone or FAP‐IR1061 NPs (100 µg mL^−1^) + Light (980 nm, 0–2.0 W cm^−2^, 3 min).

We investigated the internalization of FAP‐IR1061 in CAFs and 4T1 cells. The two cell types were cultured and then incubated with FAP‐IR1061 (Cy5 labeled) at 5.0 µm for 1, 2, 3, and 4 h. FAP‐IR1061 (Cy5 labeled) was highly internalized by both CAFs and 4T1 cells at 3 and 4 h, respectively. In addition, internalization of FAP‐IR1061 (Cy5 labeled) was greater in CAFs than in 4T1 cells at 3 h, confirming the preferential targeting of CAFs by FAP‐IR1061 (Cy5 labeled) (Figure 4A). We further examined the subcellular organelle localization of FAP‐IR1061 (Cy5 labeled) in pretreated CAFs and 4T1 cells, with results quantified using ImageJ. As illustrated in Figure 4B, a substantial amount of FAP‐IR1061 (Cy5 labeled) was internalized in CAFs. After 2 h of co‐incubation, co‐localization between FAP‐IR1061 (Cy5 labeled) (red) and lysosomes (green) was observed in pretreated CAFs and 4T1 cells, which was particularly prominent in CAFs (Figure 4C–E).

To further assess the photothermal effect of FAP‐IR1061 NPs under 980 nm laser irradiation, live/dead staining experiments and a Cell Counting Kit‐8 (CCK‐8) assay were conducted. As shown in Figure 4F, no red fluorescence was observed, but strong green fluorescence was detected in CAFs and 4T1 cells under dark conditions, indicating no significant cell damage across all the experimental groups in the absence of light. The results reflected that PTT had not been activated. In contrast, an abundant red fluorescence in both CAFs and 4T1 cells emitted from propidium iodide (PI), which binds to the DNA of dead cell, was observed after treatment with FAP‐IR1061 NPs (100 µg mL^−1^) following light exposure (980 nm, 1.0 W cm^−2^, 3 min), indicating the strong cytotoxicity of FAP‐IR1061. A CCK‐8 assay were also used to evaluate the in vitro cytotoxicity of FAP‐IR1061 NPs, yielding results consistent with the live/dead staining experiments. After a 4 h incubation of cells with FAP‐IR1061 NPs (0–200 µg mL^−1^) in the dark, the cell viabilities of CAFs and 4T1 cells both remained above 80%. In contrast, under 980 nm laser irradiation (1.0 W cm^−2^), FAP‐IR1061 NPs exhibited strong phototoxicity in a concentration‐ and laser power density‐dependent manner, with cell viability decreased to only 10% at the high concentration of 200 µg mL^−1^. Cells pretreated with FAP‐IR1061 NPs exhibited concentration‐dependent death after 3 min of laser exposure (Figure 4G,H). Additionally, an increase in laser power densities (0–2.0 W cm^−2^, 3 min) significantly reduced cell viabilities (incubated with FAP‐IR 1061 NPs, 100 µg mL^−1^) under the 980 nm laser exposure (Figure 4I,J). These distinct outcomes were attributed to the superior PTT performance of FAP‐IR1061 NPs.

### 2.5 In Vivo Imaging

**FIGURE 5:**
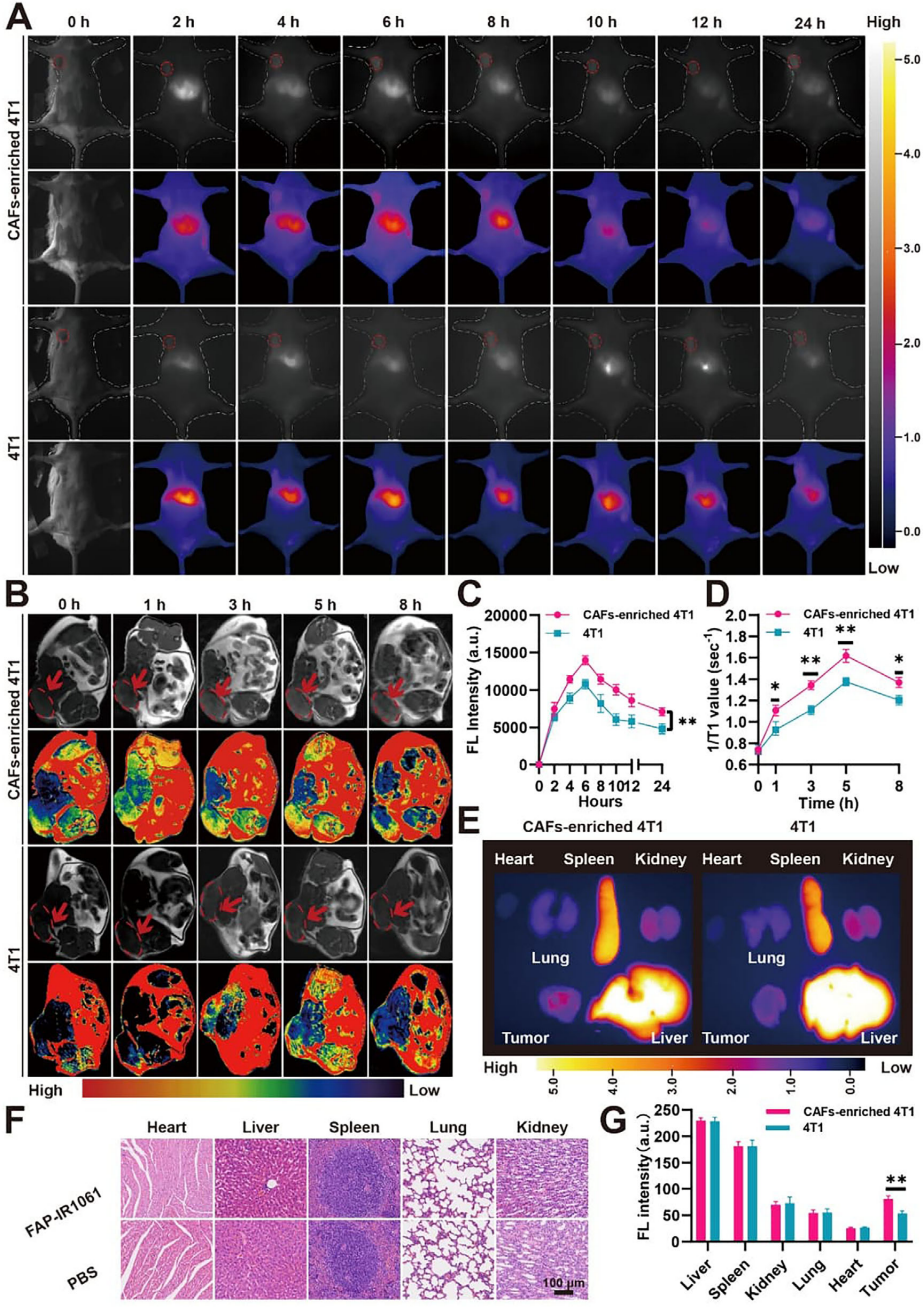
(A) NIR‐II fluorescent distribution of CAFs‐enriched 4T1 tumors and 4T1 tumors at different time points following intravenous injection of FAP‐IR1061 NPs (100 µL, 1.0 mg mL^−1^). (B) In vivo MR T1‐weighted images and pseudocolor images of mice after injection of FAP‐IR1061 NPs (100 µL, 1.0 mm). (C) In vivo NIR‐II fluorescence quantification of tumor tissues for FAP‐IR1061 NPs (100 µL, 1.0 mg mL^−1^), *n* = 5, data present as mean ± SD (*p < 0.05, **p < 0.01). (D) Time‐dependent T1 signal intensity in MR T1‐weighted images post FAP‐IR1061 NPs (100 µL, 1.0 mm), *n* = 5, data present as mean ± SD (*p < 0.05, **p < 0.01). (E) NIR‐II fluorescence quantification of tumor tissues and major organs from CAFs‐enriched 4T1 tumors and 4T1 tumors at 8 h after injection of FAP‐IR1061 NPs (100 µL, 1.0 mg mL^−1^). (F) Hematoxylin and eosin (H&E) staining images of major organs after injecting with FAP‐IR1061 NPs (100 µL, 1.0 mg mL^−1^) or PBS, scale bar: 100 µm. (G) Quantitative analysis of NIR‐II fluorescence intensity of FAP‐IR1061 NPs (100 µL, 1.0 mg mL^−1^) in tumors tissues and major organs, *n* = 5, data present as mean ± SD (**p < 0.01).

To evaluate improved tumor retention and biodistribution, in vivo NIR II, MR, and PA imaging were conducted on tumor‐bearing mice divided into two groups (*n*  =  5): (1) mice with 4T1 tumors and (2) mice with CAFs‐enriched 4T1 tumors. Mice with tumors measuring around 100–200 mm^3^ received an intravenous tail vein injection of 100 µL (1.0 mg mL^−1^) FAP‐IR1061 NPs. Subsequently, the tumors were imaged via NIR‐II imaging (1000–1100 nm region) at 2, 4, 6, 8, 10, and 24 h postinjection. As shown in Figure 5A–D, mice bearing CAFs‐enriched 4T1 tumors administrated FAP‐IR1061 NPs exhibited a marked enhancement in NIR‐II (100 µL, 1.0 mg mL^−1^) and MR T1‐weighted imaging signals (100 µL, 1.0 mm) within the tumors, which peaked at 6 h postinjection. In contrast, a much weaker fluorescence increase was observed at 4T1 tumor sites of mice injected with FAP‐IR1061 NPs at the same time points. For the two groups treated with nontargeted IR1061 NPs, no significant difference in NIR‐II imaging signals was observed between the two groups within the first 6 h. However, at later time points (8–12 h), the signal intensity in 4T1‐only tumors peaked and became significantly higher than that in CAFs‐enriched tumors, reflecting the enhanced barrier function of CAFs‐enriched stroma against nontargeted IR1061 NPs (Figure S5A,B). To assess organ distribution in tumor‐bearing mice, tumors and major organs were excised at 8 h postinjection, and their fluorescence intensity was evaluated. According to Figure 5E–G and Figure S5C–E, the livers and spleens of the mice showed enhanced NIR‐II fluorescence signals, suggesting that FAP‐IR1061 NPs were predominantly metabolized in these organs.

FAP‐IR1061 NPs can also serve as a PA contrast agent. As illustrated in Figure S6A, in vivo PA imaging was subsequently performed on tumor‐bearing mice. Tumor signal intensity in CAFs‐enriched 4T1 tumors increased gradually, reaching a maximum value at 6 h postinjection. To quantitatively measure the signal intensities associated with tumor retention, PA signal intensity was measured and presented in Figure S6B. As depicted in Figure S6B, the PA signal intensity of the CAFs‐enriched 4T1 breast cancer model group was stronger than that of the standard 4T1 solid tumor model at 6 h. Collectively, the multimodal approach of NIR‐II, MR, and PA imaging improves the imaging accuracy for CAFs‐enriched 4T1 tumors.

Altogether, the hemolytic activity of FAP‐IR1061 NPs was further evaluated to determine its potential to cause acute hemolysis when directly exposed to blood. Mouse erythrocytes were exposed to different concentrations of FAP‐IR1061 NPs (100 µL, 1.0 mg mL^−1^), and hemoglobin release was determined by measuring absorbance at 540 nm. After a 24 h incubation, the positive control group employing deionized distilled water (ddH_2_O) as the medium for erythrocyte dispersion, exhibited notable hemolysis, indicated by a red supernatant. However, no significant hemolytic reactions were observed in the FAP‐IR1061 NPs group, confirming their blood compatibility. (Figure S6C). Finally, measurements of alanine aminotransferase (ALT), aspartate aminotransferase (AST), alkaline phosphatase (ALP), urea (UREA), and creatinine (CREA) in mice fell within normal parameters, suggesting that the administrations of FAP‐IR1061 NPs had minimal effects on liver and kidney functions. These results demonstrate that the impact of FAP‐IR1061 NPs on normal organs is negligible, indicating low systemic toxicity (Figure S6D). Biodistribution of Mn was evaluated in both tumor‐bearing and healthy mice. In two types of tumor‐bearing mice, Mn levels in major organs and tumors were assessed 24 h after injection of FAP‐IR1061 NPs (100 µL, 1.0 mm), revealing predominant accumulation in the kidneys with no significant difference between the two models. In healthy mice, a time‐course study showed that Mn levels in the blood began to rise at 1 h postinjection, peaked at 2 h, and gradually decreased thereafter. In organs, Mn primarily accumulated in the heart, liver, lungs, and kidneys, with the highest levels observed in the livers and kidneys, reaching peak concentrations at 6–8 h postinjection and clearing by 24 h, consistent with urinary excretion, while fecal Mn levels remained unchanged throughout the time course (Figure S6E–I and Table S1).

### 2.6 The Photothermal Properties of FAP‐IR1061 Nanoparticles Inhibit the Proliferation and Migration of CAFs in 4T1 Cells

**FIGURE 6:**
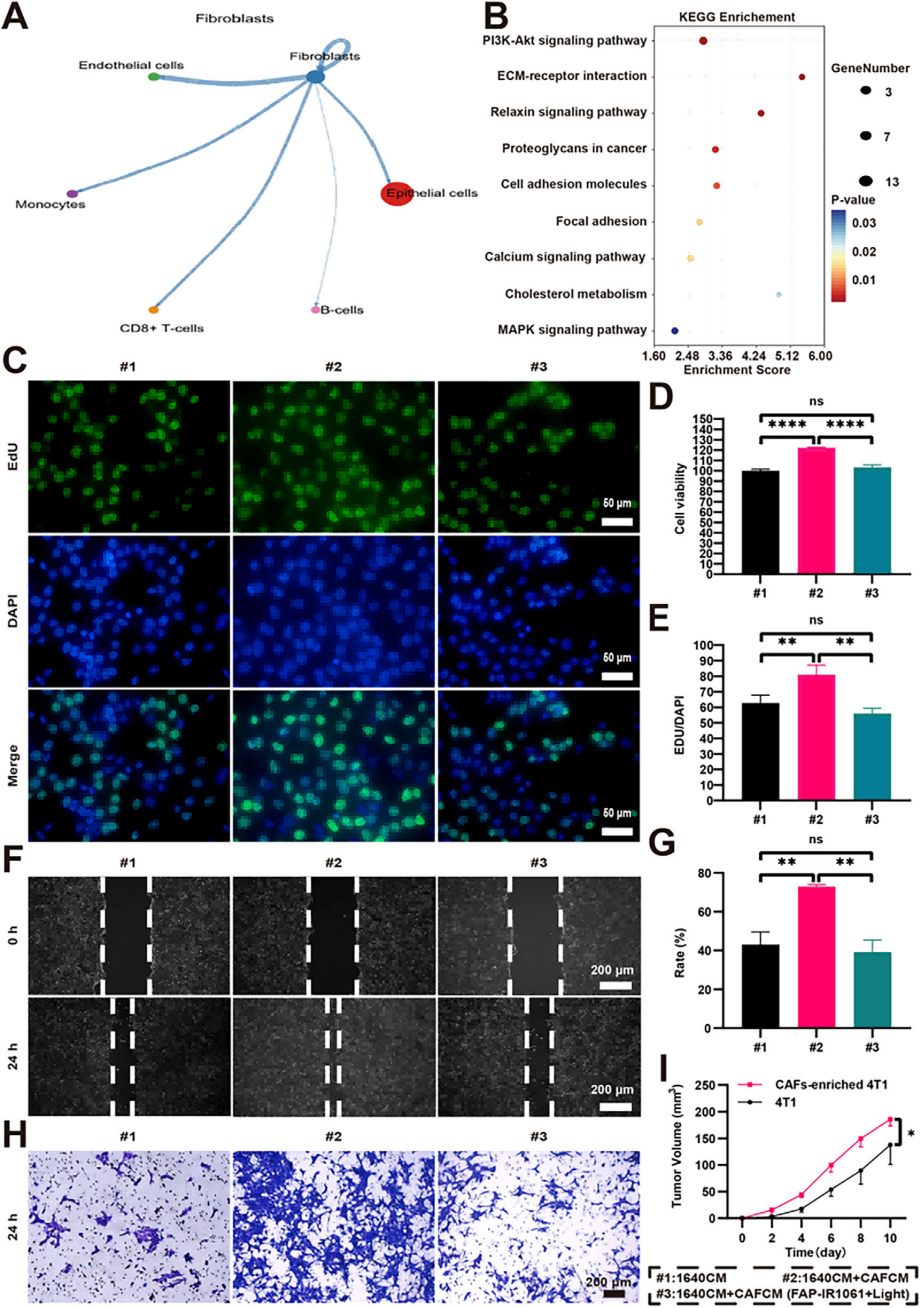
(A) CellChat analysis of fibroblasts. (B) KEGG pathway enrichment analysis of 4T1 cells and CAFs‐regulated 4T1 cells. (C–H) EdU assay (C,E), CCK‐8 assay (D), scratch wound healing assay (F,G), and Transwell assay (H) of 4T1 cells cocultured with conditioned medium (CM) derived from CAFs, either before or after PTT treatment of FAP‐IR1061 NPs (100 µg mL^−1^, 980 nm, 1.0 W cm^−2^, 3 min). EdU is in the green channel (*λ*_ex_ = 488 nm, *λ*_em_ = 500–550 nm), DAPI is in the blue channel (*λ*_ex_ = 405 nm, *λ*_em_ = 450–500 nm) (** *p* ＜ 0.01, **** *p* ＜ 0.0001). (I) Tumor volume change curves of mice in the CAFs‐enriched 4T1 tumor model and the 4T1 tumor model, *n* = 5, data are presented as mean ± SD (*p < 0.05).

Cellchat analysis revealed that fibroblasts primarily interact with tumor cells, vascular endothelial cells, macrophages, and CD8^+^ T cells [8] (Figure 6A). Previous studies had demonstrated that CAFs in the TME enhance cancer cell proliferation and migration [14, 52]. Transcriptome sequencing results showed that key signaling pathways such as PI3K‐AKT, MAPK, and TGF‐β were enriched in 4T1 cells upon CAFs coculture, suggesting their potential role in driving cell proliferation and migration (Figure 6B). In order to evaluate whether the photothermal properties of FAP‐IR1061 NPs can reverse CAF‐induced proliferation and migration of 4T1 cells, CCK‐8 assay, EdU assay analysis, scratch wound healing assay and Transwell assay were performed in vitro. The experimental results showed that when 4T1 cells were cocultured with CAFs‐derived CM, CAFs promoted their proliferation and migration. However, the proliferation and migration of 4T1 cells were suppressed when cocultured with CM from CAFs following FAP‐IR1061 NPs‐mediated PTT (100 µg mL^−1^, 980 nm, 1.0 W cm^−2^, 3 min) (Figure 6C–H). Additionally, tumors in CAFs‐enriched 4T1 mice exhibited a faster growth rate (Figure 6I). These results demonstrate intricate crosstalk between CAFs and the TME in TNBC.

### 2.7 Photothermal Therapy can Directly Remodel ECM and Induce ICD of 4T1 Cells In Vitro

**FIGURE 7:**
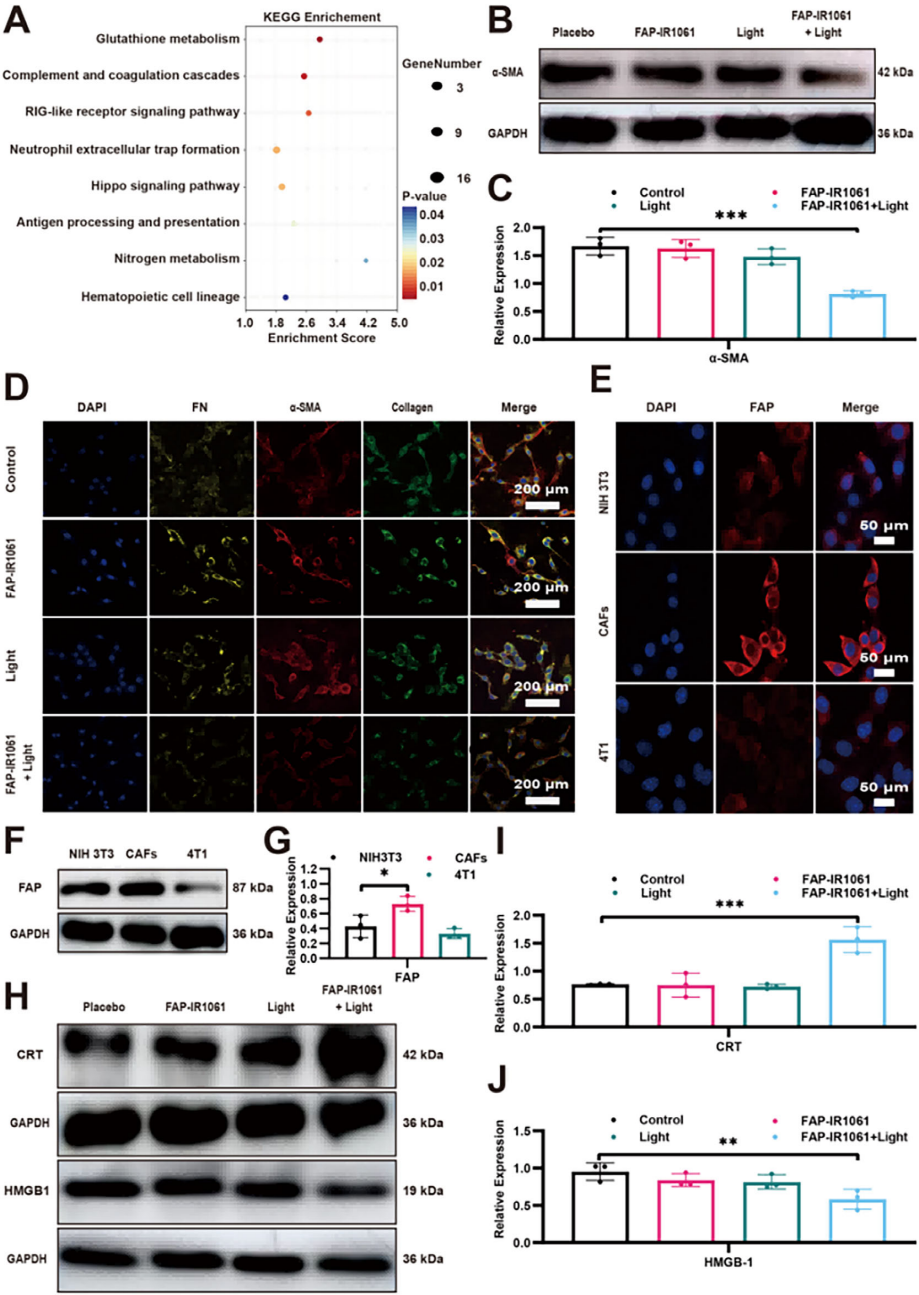
(A) KEGG pathway analysis of PTT­regulated oxidative stress in CAFs (100 µg mL^−1^, 980 nm, 1.0 W cm^−2^, 3 min). (B,C) Western blot analysis of α‐SMA in CAFs after treatments with Control, FAP‐IR1061 NPs (100 µg mL^−1^), Light (980 nm, 1.0 W cm^−2^, 3 min), and FAP‐IR1061 NPs (100 µg mL^−1^) + Light (980 nm, 1.0 W cm^−2^, 3 min), respectively. *n* = 3, data are presented as mean ± SD (*** *p* < 0.001). (D) Immunofluorescence experiments of ECM‐related proteins in CAFs after treatments with Control, FAP‐IR1061 NPs (100 µg mL^−1^), Light (980 nm, 1.0 W cm^−2^, 3 min), and FAP‐IR1061 NPs (100 µg mL^−1^) + Light (980 nm, 1.0 W cm^−2^, 3 min), respectively. α‐SMA: red channel, *λ*_ex_ = 640 nm, *λ*_em_ = 655–750 nm; FN: yellow channel, *λ*_ex_ = 561 nm, *λ*_em_ = 570–620 nm; and collagen: green channel, *λ*_ex_ = 488 nm, *λ*_em_ = 500–550 nm; DAPI: blue channel, *λ*_ex_ = 405 nm, *λ*_em_ = 450–500 nm. (E–G) Western blot analysis and immunofluorescence experiments of FAP in 4T1 cells, respectively. FAP: red channel, *λ*_ex_ = 561 nm, *λ*_em_ = 600–650 nm; DAPI: blue channel, *λ*_ex_ = 405 nm, *λ*_em_ = 450–500 nm. *n* = 3, data are presented as mean ± SD (* *p* < 0.05). (H–J) Western blot analysis of CRT and HMGB1 in 4T1 cells after treatments with Control, FAP‐IR1061 NPs (100 µg mL^−1^), Light (980 nm, 1.0 W cm^−2^, 3 min), and FAP‐IR1061 NPs (100 µg mL^−1^) + Light (980 nm, 1.0 W cm^−2^, 3 min), respectively. *n* = 3, data are presented as mean ± SD (** *p* < 0.01, and *** *p* < 0.001).

PTT targeting CAFs (incubated with FAP‐IR 1061 NPs, 100 µg mL^−1^, 980 nm, 1.0 W cm^−2^, 3 min) can induce oxidative stress in CAFs and inhibit their pro‐cancer effects by remodeling the TME, reducing the expression of ECM proteins, enriching the Hippo signaling pathway, modulating immune responses and metabolic regulation (Figure 7A). In vitro GSEA results revealed that multiple pathways were significantly upregulated, including antigen presentation, TLR signaling pathways, stress response‐related pathways, and Neutrophil Extracellular Trap (NET) formation. In contrast, pathways involved in DNA repair, including cell cycle regulation, DNA replication, mismatch repair, and homologous recombination, were significantly downregulated (Figure S7). These data systematically explain the intrinsic changes in the function and molecular mechanisms of CAFs following PTT treatment in vitro. WB and mIF experiments further confirmed that the expression of α‐SMA and ECM‐related proteins on the surface of CAFs decreased after PTT (incubated with FAP‐IR 1061 NPs, 100 µg mL^−1^, 980 nm, 1.0 W cm^−2^, 3 min) (Figure 7B–D).

Previous fractionation and FAP visualization based on single‐cell data from GEO CAFs (GSE199515) indicated that FAP was also expressed at low levels on epithelial cells (Figure S2). WB and immunofluorescence result also showed low FAP expression on the surface of 4T1 cells (Figure 7E,F). Thus, FAP‐IR1061 NPs can also directly target 4T1 cells. When PTT is initiated (incubated with FAP‐IR 1061 NPs, 100 µg mL^−1^, 980 nm, 1.0 W cm^−2^, 3 min), the ICD is triggered, releasing DAMPs to activate the immune system. Typical ICD biomarkers, such as CRT and HMGB 1, were measured to study the immune response induced by FAP‐IR1061 NPs. CRT expression on the 4T1 cell membrane was obviously upregulated, while the intracellular HMGB1 was greatly downregulated (Figure 7G–J).

### 2.8 PTT Combined with ICB Therapy to Inhibit Fibrotic Breast Cancer

**FIGURE 8:**
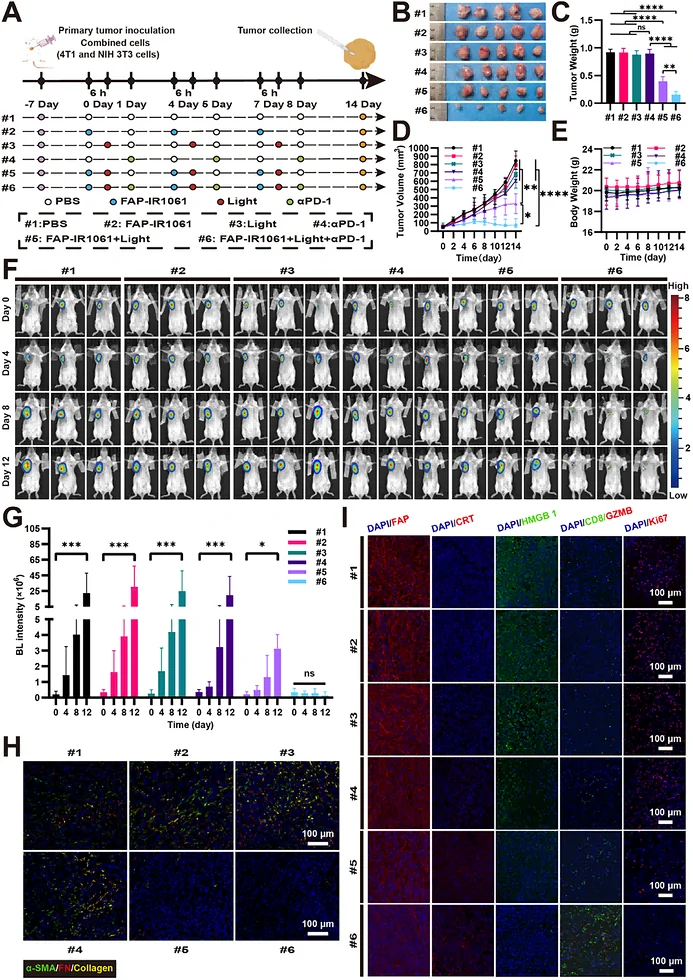
Treatment of primary fibrotic breast cancer with PTT and αPD‐1. (A) Depiction of CAFs‐enriched 4T1 primary tumor inoculation and the corresponding therapeutic strategies in BALB/c mice. (B–D) Representative images (B), weight of tumors (C), and volume change curves (D), *n* = 6, data present as mean ± SD (*p < 0.05, **p < 0.01, and ****p < 0.0001). (E) Body weights of mice in all groups. (F) Bioluminescence (BL) imaging of the treatment response in BALB/c mice. (G) Quantified tumor BL intensities of the treatment response in BALB/c mice, *n* = 6, data present as mean ± SD (*p < 0.05, and ***p < 0.001). (H) mIF images of α‐SMA, FN, Collagen in tumors after different treatments, scale bar: 100 µm. FN: red channel, *λ*_ex_ = 640 nm, *λ*_em_ = 655–750 nm; collagen: yellow channel, *λ*_ex_ = 561 nm, *λ*_em_ = 570–620 nm; and α‐SMA: green channel, *λ*_ex_ = 488 nm, *λ*_em_ = 500–550 nm; DAPI: blue channel, *λ*_ex_ = 405 nm, *λ*_em_ = 450–500 nm. (I) Immunofluorescence images of FAP, CRT, HMGB 1, granzyme B‐positive (GZMB^+^) CTLs, CD8^+^ CTLs, and Ki‐67 in tumors after different treatments, FAP, CRT, GZMB^+^ CTLs, Ki‐67: red channel, *λ*_ex_ = 561 nm, *λ*_em_ = 600–650 nm; HMGB 1, CD8^+^ CTLs: green channel, *λ*_ex_ = 488 nm, *λ*_em_ = 500–550 nm; DAPI: blue channel, *λ*_ex_ = 405 nm, *λ*_em_ = 450–500 nm. Scale bar: 100 µm.

Given the promising in vitro antitumor results and increased in vivo tumor retention, we proceeded to evaluate the therapeutic efficacy of various treatments in vivo. As shown in Figure 2F, a CAFs‐enriched 4T1 breast cancer model was developed as a primary tumor model using xenografts of luciferase‐expressing 4T1 cells. Drug administration and PTT followed the schedule outlined in Figure 8A. To be specific, six groups (*n* = 6) of mice bearing CAFs‐enriched 4T1 tumor were treated as follows: PBS, FAP‐IR1061 (100 µL, 1.0 mg mL^−1^), Light irradiation alone (3 min at 1.0 W cm^−^^2^), αPD‐1 (5 mg kg^−1^), FAP‐IR1061 (100 µL, 1.0 mg mL^−1^) + Light irradiation (3 min at 1.0 W cm^−^^2^), and FAP‐IR1061 (100 µL, 1.0 mg mL^−1^) + Light (3 min at 1.0 W cm^−^^2^) + αPD‐1 (5 mg kg^−1^), respectively (Figure 8A).

Over a 14‐day period, mice were observed for alterations in body weight and tumor volume, with samples collected at the endpoint for tumor analysis (Figure 8B–E). The stability in weight throughout PTT and ICB treatment indicates the safety of these interventions. Hematoxylin and eosin (H&E) staining major organs, such as the heart, liver, spleen, lungs, and kidneys, were analyzed for cell morphology. No obvious organ lesions were observed in all groups, indicating that these treatments have a negligible influence on normal organs (Figure 8C and Figure S8A). Compared to the PBS group, mice in the single αPD‐1 group exhibited similarly rapid tumor growth. In contrast, FAP‐IR1061 + Light group showed slight tumor suppression, suggesting that PTT alone exerted a moderately delayed therapeutic effect. Notably, the combination of PTT and αPD‐1 demonstrated the most effective antitumor response, achieving approximately 83% tumor inhibition, highlighting the superiority of the combined therapy (Figure 8C–E). Simultaneously, a reduction in spleen size and volume in the FAP‐IR1061 + Light + αPD‐1 group indicated effective alleviation of tumor‐induced splenomegaly (Figure S8B). Real‐time bioluminescence (BL) imaging in BALB/c mice was used to assess the treatment response (Figure 8F,G). Quantified tumor BL images and intensities indicated delayed tumor proliferation in the FAP‐IR1061 + Light + αPD‐1 group, compared to rapid growth observed in the other groups. As shown in Figure 8H,I and Figure S8C, combined treatment inhibited FAP expression in tumor tissues. Furthermore, α‐SMA that indicated the activation of CAFs, was also inhibited. Additionally, the level of FN and Collagen proteins were also downregulated following PTT combined with ICB Therapy. Therefore, these results indicate that PTT can reprogram CAFs and reshape the ECM in TME. RNA sequencing (RNA‐Seq) yields similar result. FAP‐IR1061 + Light partially disrupted the physical barrier formed by CAFs and ECM, the disruption remained incomplete. Moreover, due to the inherent role of CAFs in wound repair, PTT triggered feedback upregulation of ECM‐receptor interaction and cell adhesion molecules (Figure S9A–C). In contrast, αPD‐1 immunotherapy failed to effectively disrupt the physical barrier and even resulted in increased focal adhesion, potentially promoting tumor invasion and metastasis, which is a possible mechanism underlying treatment failure (Figure S9C,D). Notably, compared with PBS group, the FAP‐IR1061 + Light group, and the αPD‐1 group, the combination therapy significantly reduced ECM deposition, successfully targeting both CAFs and ECM and thoroughly disrupting the physical barrier, as evidenced by the downregulation of pathways including ECM‐receptor interaction, focal adhesion, cell adhesion molecules, regulation of actin cytoskeleton, and osteoclast differentiation (Figure S10A–D).

### 2.9 In Vivo ICD and Immune Response Induced by Combined Treatment

PTT has been shown to trigger ICD and release DAMPs to activate the immune system. As mentioned above, typical ICD markers like CRT, and HMGB 1 had been assessed in 4T1 cells to examine the immune response caused by FAP‐IR1061 NPs (Figure 7G–J). These findings were further confirmed in animal experiments. Among all treatment groups, FAP‐IR1061 + Light + αPD‐1 showed the highest CRT expression and the lowest HMGB 1 levels (Figure 8I and Figure S8D,E). Additionally, PTT + αPD‐1‐treated tumors had significantly lower Ki‐67 expression and stronger TUNEL staining signals than other groups, indicating markedly reduced tumor cell proliferation and obvious apoptosis (Figure 8I and Figure S8F–H).

The ECM can restrict drug penetration and impede immune cell infiltration, thus increasing immune resistance [10, 35]. Our cellular and animal experiments had demonstrated that FAP‐IR1061 NPs + PTT + αPD‐1exert potent ECM reshaping capability in the TME upon light exposure. Therefore, it was necessary to further assess the depth of infiltration of immune cells in CAFs‐enriched 4T1 breast cancer after different treatment. Tumor‐infiltrating lymphocytes (TILs) serve as a predictive biomarker for ICB response [53, 54, 55]. Interestingly, the combination of PTT and αPD‐1 increased the infiltration of CD8‐positive CTLs (CD8^+^ CTLs) and granzyme B‐positive CTLs (GZMB^+^ CTLs), demonstrating robust systemic immune activation and improved TILs penetration through the ECM barrier to reach deeper tumor region (Figure 8I and Figure S8I,J). Nevertheless, when PTT or αPD‐1 therapy is used alone, CD8^+^ CTLs and GZMB^+^ CTLs cannot be effectively activated or delivered to the tumor site as in the combined therapy group. The results of Bulk RNA‐Seq further elaborate on the underlying mechanisms of incomplete immune activation induced by PTT or αPD‐1 therapy alone. ICD induced by PTT activated alarm signals, as evidenced by the upregulation of antigen processing and presentation [56, 57]. In addition, the upregulation of Th17/Th1/Th2 differentiation [58], and Inflammatory mediator regulation of Transient Receptor Potential (TRP) channels [59] indicated that PTT also fostered a dysregulated TME characterized by chronic inflammation. Critically, these responses failed to effectively activate T cells, thereby preventing thorough tumor clearance. In response, a state of primary immunodeficiency emerged, reflecting the ultimate immune imbalance (Figure S9A,B). Similarly, while αPD‐1 therapy enhanced antigen presentation [56, 57], it did not elicit substantial antitumor immunity (Figure S9C,D). These findings demonstrate that neither PTT alone nor αPD‐1 therapy can induce significant antitumor immune effects, merely exerting an immune warning function. In contrast, the combination therapy markedly reduced protumorigenic cytokine signaling, as evidenced by downregulation of cytokine–cytokine receptor interaction, while activating humoral immunity through concurrent enrichment of B cell receptor signaling (Figure S10A,B). On this basis, the combination therapy effectively downregulated TGF‐β, a key regulatory factor that promotes fibrosis and immunosuppression [13, 60, 61], further disrupted the ECM, creating conditions for the infiltration of T cells and other immune cells into tumor tissues (Figure S10A–D). Additionally, the combination therapy downregulated HIF‐1α [41] to ameliorate hypoxia and reversed functional suppression of T cells, as demonstrated by the downregulation of the PD‐L1/PD‐1 checkpoint pathway [37, 42, 62], Toll‐like receptor signaling pathway [63, 64], and NOD‐like receptor signaling pathway [65] (Figure S10B–D). Collectively, these changes reprogramed the TME from a chronic inflammatory state toward an effective T cell‐mediated antitumor response.

**FIGURE 9:**
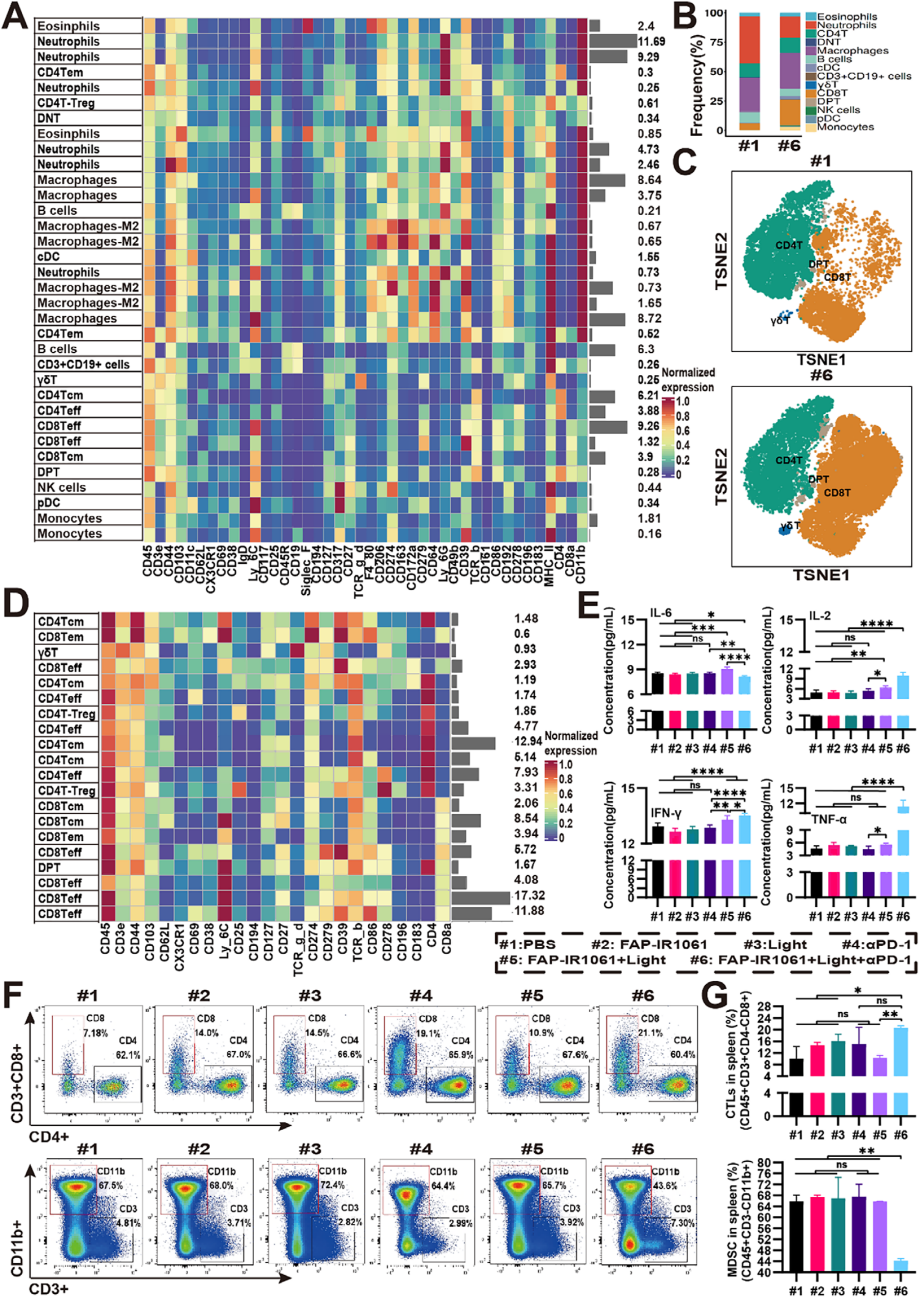
ICD activation and immune cell infiltration. (A) Heatmap to visualize total immune cells in the TME. (B) The distribution of the main immune cell types in the TME. (C) T‐distributed stochastic neighbor embedding (TSNE) graphs of T cells. (D) Heatmap to visualize T cells in the TME. (E) Relative concentration of IL‐6, IL‐2, IFN‐γ, and TNF‐α by ELISA, *n* = 6, data present as mean ± SD (*p < 0.05, **p < 0.01, ***p < 0.001, and ****p < 0.0001). (F,G) Flow cytometric analysis (gated on CD45^+^) and quantification of cytotoxic T cells (CTLs) marked with CD45^+^CD3^+^CD4^−^CD8^+^ and myeloid‐derived suppressor cells (MDSCs) marked with CD45^+^CD3^−^CD11b^+^. *n* = 6, data present as mean ± SD (*p < 0.05, and **p < 0.01).

To comprehensively evaluate the immune activation by combined PTT and ICB therapy, immune cell infiltration in the TME was further analyzed. High‐dimensional mass spectrometry flow cytometry (CyTOF) was used to characterize TILs, which were classified into 34 groups according to 42 immune markers (Figure 9A and Figure S11A–C). In the FAP‐IR1061 + Light + αPD‐1 group, the overall expression of CD38 and CD86 on immune cells was significantly upregulated, indicating activation of lymphocytes and antigen‐presenting cells (APCs) (Figure S11B). Consistently, DCs, especially for conventional DCs (cDCs), were significantly increased in the FAP‐IR1061 + Light + αPD‐1 group compared with the PBS group (Figure 9B and Figure S11D). Additionally, the number of CD8^+^ T cells, were also significantly increased in the FAP‐IR1061 + Light + αPD‐1 group. T cells were clustered into 20 subsets based on 24 immune markers (Figure 9C,D and Figure S11E). Notably, the proportion of CD8^+^ effector T cells (CD8Teff) in the FAP‐IR1061 + Laser + αPD‐1 group was significantly higher than that in the PBS group (Figure S11F). As shown in Figure 9B, neutrophils were significantly reduced in the FAP‐IR1061 + Light + αPD‐1 group. Therefore, the neutrophil subgroups in the TME were further analyzed. Neutrophils were divided into 16 subsets (C01‐16) according to 19 immune markers (Figure S11G), with total neutrophil numbers significantly lower in the FAP‐IR1061 + Light + αPD‐1 group than in the PBS group (Figure S11G). Following PTT‐immunotherapy, we observed a significant activation of neutrophils within the TME as evidenced by the upregulated expression of activation markers, including CD38, CD86, CD11b, and Ly‐6C (Figure S11H). Further high‐dimensional single‐cell analysis revealed profound reshaping of the neutrophil landscape. Specifically, the frequencies of three neutrophil subsets (C09, C10, and C12) were markedly decreased, while the subset of C15 was concurrently expanded (Figure S11J), suggested a phenotypic shift from protumorigenic to antitumorigenic states. Notably, TANs, one subset of myeloid‐derived suppressor cells (MDSCs), have been well‐recognized as “double‐edged swords” in the TME [39, 66]. N1 subtype primarily exerts antitumor effects, whereas the N2 subtype exhibits prominent protumor properties [38, 39, 66, 67]. Given this, the concurrent reduction in C09/C10/C12 and expansion of C15 implied that the combination therapy might selectively deplete N2‐like immunosuppressive neutrophil subsets (e.g., C09/C10/C12) while enriching N1‐like immunostimulatory subsets (e.g., C15). Whereas depleted subsets may represent immunosuppressive populations whose elimination is critical for a successful antitumor immunity. These findings demonstrated that PTT‐immunotherapy combination therapy does not simply alter the number of neutrophils or induce their activation, but rather drives neutrophil subsets to undergo precise and critical repolarization reprogramming. We further confirmed the phenotypic reprogramming of neutrophils using RNA‐Seq. The downregulation of NET [38, 66, 67] formation in the FAP‐IR1061 + Light + αPD‐1 group (Figure S10B), consistent with the results from CyTOF. NETs capture circulating tumor cells (CTCs) in the bloodstream to promote distant colonization [68]. Additionally, NETs directly suppress the antitumor activity of T cells and NK cells [38]. The downregulation of NETs should be interpreted as evidence of functional reprogramming in the TAN population within the TME, suggesting strong potential for these therapies to suppress tumor invasion and metastasis.

The impact of PTT and αPD‐1 on immune modulation was also evident in vivo and cytokine levels (Figure 9E). The result of enzyme‐linked immunosorbent assay (ELISA) kits showed that CAFs suppress the immune system by releasing large quantities of IL‐6, which impairs immune cell function [13]. Nonetheless, PTT combined with ICB therapy reduced IL‐6 secretion. Moreover, IL‐2 and Interferon‐γ (IFN‐γ) expressions, which are signs of T cell activation and enhanced T cell cytotoxicity respectively [69], were elevated. Relative levels of circulating cytokines Tumor Necrosis Factor α (TNF‐α) in the FAP‐IR1061 + Laser + αPD‐1 group were approximately 1.8 folds higher than those in the PBS group, indicating a robust antitumor immune response [69] (Figure 9E). Immune cell infiltration in the spleen also confirmed the activation of cytotoxic CD8^+^ T cells and the alleviation of the immunosuppressive TME. As shown in Figure 9F,G, CD45^+^CD3^+^CD4^−^CD8^+^ CTLs were increased in the spleen while CD45^+^CD3^−^CD11b^+^ MDSCs were decreased in the FAP‐IR1061 + Light + αPD‐1 group. These results revealed that combination therapy reprogrammed the TME as well as the spleen in mice, fostering antitumor immunity by enhancing CD8^+^ T cell activation and expansion while suppressing MDSCs, aligning with the reduction in tumor‐induced splenomegaly (Figure S8B). All the above results demonstrate that the excellent ICD and PTT efficacy can indeed alleviate the natural barrier of ECM, facilitate immune activation, and improve immune cell infiltration.

Regarding whether the tumor develops feedback‐mediated resistance during these treatments, RNA‐Seq results provided an answer. PTT directly‐induced tumor cell death, causing DNA damage and significant alterations in the permeability and function of local tumor vasculature. This imposed substantial survival pressure on tumor cells, prompting their transition to a vulnerable state characterized by upregulated calcium signaling pathways, cAMP signaling pathways, and cGMP‐PKG signaling pathways [70, 71, 72] (Figure S9A,B). Furthermore, PTT elicited intense local inflammation and tumor vascular dysfunction, as evidenced by enhanced inflammatory mediator regulation of TRP channels [59] and vascular smooth muscle contraction [73] (Figure S9A,B). Under such circumstances, tumor cells exhibited enhanced repair capacity, as evidenced by upregulated processes such as DNA replication [74, 75] (Figure S9B). The observed upregulation of the Ras and mTOR signaling pathways [41] reflects a profound, multilayered feedback resistance mechanism in tumor cells, representing a more aggressive and therapeutically challenging adaptive response (Figure S9B). Concurrently, metabolic activity was elevated via the upregulation of key pathways such as glycolysis, gluconeogenesis, glycine, serine, and threonine metabolism [41, 76] (Figure S9B). In the αPD‐1 group, while αPD‐1 activated T cells, it failed to breach the physical barrier in CAFs‐enriched 4T1 breast cancer. Furthermore, it elicited tumor stress and potent adaptive resistance, as demonstrated by upregulation of DNA replication, cell cycle [74, 75, 77], PI3K‐Akt signaling pathways, MAPK signaling pathways, and mTOR signaling pathways [41, 77] (Figure S9C,D). Ultimately, the overwhelming metabolic stress from uncontrolled proliferation triggers the collapse of the protein synthesis and processing system [78, 79], as indicated by the marked downregulation of key processes including protein processing in the endoplasmic reticulum, protein export, ribosome, and protein digestion and absorption both in the FAP‐IR1061 + Light group and the αPD‐1 group (Figures S9A,C, and S12A,B). In contrast, compared with the PBS group, the FAP‐IR1061 + Light group, and the αPD‐1 group, PI3K‐Akt signaling pathways and MAPK signaling pathways [41, 77] were effectively suppressed, thereby blocking tumor growth and tumor cell resistance to apoptosis induced by combining PTT and αPD‐1 (Figure S10A–D). Moreover, CAFs‐mediated enhancement of tumor cell viability, migration, and proliferation via the JAK‐STAT signaling pathway [13] was the correspondingly suppressed (Figure S10B). Concurrently, certain high‐stress states were alleviated, as evidenced by downregulation of cAMP signaling pathways, and cGMP‐PKG signaling pathways [71, 72] (Figure S10A,B). Furthermore, tumor vessel normalization at the treatment site was observed, specifically manifested as the downregulation of vascular smooth muscle contraction [73], VEGF signaling pathways [42, 80, 81, 82, 83], and HIF‐1 signaling pathways [42, 62, 80, 81, 82] (Figure S10A,B). A small subset of residual tumor cells actively resisted therapeutic stress by sustaining DNA repair characterized by upregulated DNA repair and replication pathways including DNA replication, cell cycle, homologous recombination, nucleotide excision repair, and base excision repair [74, 75, 84] (Figure S12C). Such a sequence of events led tumors to maintain a hypermetabolic profile, characterized by enhanced metabolic pathways including oxidative phosphorylation, the TCA cycle, thermogenesis, fatty acid degradation, ribosome biogenesis, proteasome activity, pyrimidine metabolism, and glutathione metabolism [41, 76, 78] (Figure S12C–E).

In summary, the combined therapy exerted its effects by remodeling the ECM, activating the T cell‐mediated tumor killing effect, and blocking the therapeutic resistance of tumor cells. Interestingly, while KEGG analysis of cells treated with FAP‐IR1061 + Light in vitro revealed enrichment of the NET formation pathway (Figure 7A), and further GSEA analysis (Figure S7A) confirmed NET upregulation. GSEA analysis of tumor tissues from mice in FAP‐IR1061 + Light group showed partial inconsistency, including downregulation of NET‐related pathways (Figure S12A), suggesting that the complex in vivo immune microenvironment, which involving multiple cell types and regulatory networks, leads to a distinct integrated outcome. Notably, a significant common effect across mice from all treatment groups was the observed downregulation of NET formation (Figures S10B and S12A,B), indicating considerable potential of these treatments in inhibiting tumor invasion and metastasis. However, whether these treatments could consistently suppress tumor recurrence and metastasis remains unclear and requires further experimental validation.

### 2.10 PTT Combined with ICB Therapy to Sensitize Therapy of the Recurrent Breast Cancer

**FIGURE 10:**
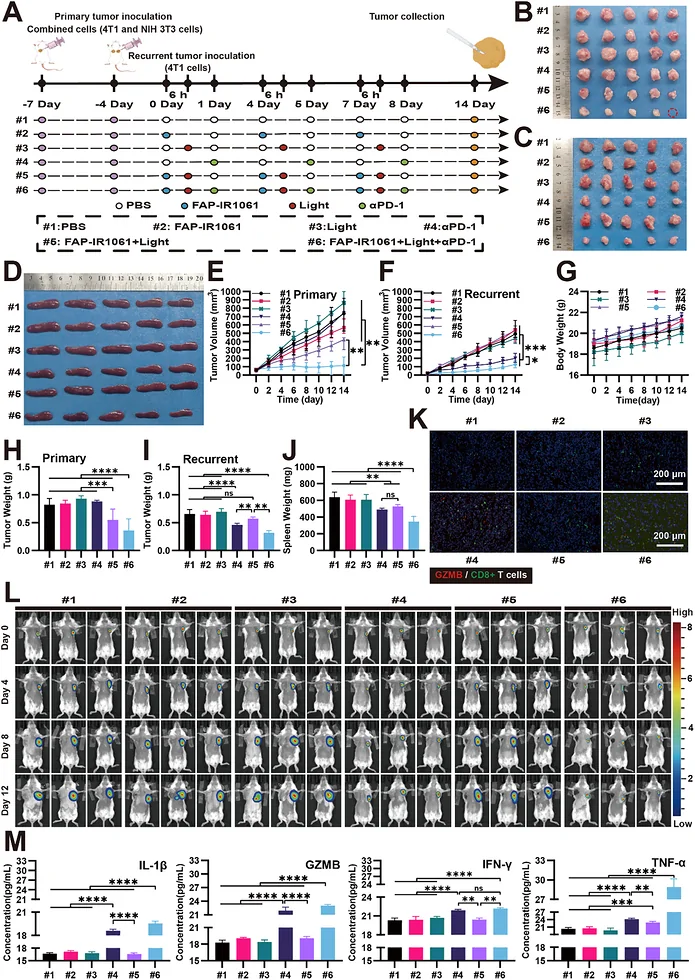
Treatment of primary fibrotic breast cancer and recurrent breast cancer with PTT and αPD‐1. (A) Timeline schematic of CAFs‐enriched 4T1 primary tumors and 4T1 recurrent distant tumors inoculation, along with therapeutic strategies in BALB/c mice. (B–D) Representative images of primary tumors, recurrent tumors, and spleen in all groups. (E,F) Volume change curves of primary tumors and recurrent tumors, *n* = 6, data present as mean ± SD (*p < 0.05, **p < 0.01, and ***p < 0.001). (G) Body weights in all groups. (H–J) Weight of primary tumors, recurrent tumors, and spleen, *n* = 6, data present as mean ± SD (**p < 0.01, ***p < 0.001, and ****p < 0.0001). (K) Immunofluorescence images of CD8^+^ CTLs and GZMB^+^ CTLs in recurrent tumors after different treatments. GZMB^+^ CTLs: red channel, *λ*_ex_ = 561 nm, *λ*_em_ = 600–650 nm; CD8^+^ CTLs: green channel, *λ*_ex_ = 488 nm, *λ*_em_ = 500–550 nm; DAPI: blue channel, *λ*_ex_ = 405 nm, *λ*_em_ = 450–500 nm. Scale bar: 200 µm. (L) BL imaging of the treatment response in BALB/c mice. (M) Relative concentration of IL‐1β, GZMB, IFN‐γ, and TNF‐α by ELISA, *n* = 6, data present as mean ± SD (**p < 0.01, ***p < 0.001, and ****p < 0.0001).

To confirm the therapeutic potential of FAP‐IR1061‐Laser combined with ICB for treating relapsed tumors, a bilateral tumor model was established, as illustrated in Figure 10A. The CAFs‐enriched 4T1 breast cancer model was initially developed as a primary tumor, and three days later, 4T1‐Luc cells were introduced to the left side as recurrent tumors to evaluate the anti‐recurrence efficacy of the regimen by monitoring tumor regrowth dynamics. PTT and therapeutic interventions were executed as planned for the primary tumor, and volumetric alterations in both the primary and relapsed tumors were monitored over a 14‐day period. Over 14 days, mice were observed for alterations in body weight and tumor volume, with samples taken at the end of treatment for tumor examination (Figure 10B–G). As demonstrated in Figure 10B,C,E,F,H,I, bilateral tumors in the PBS, FAP‐IR1061 (100 µL, 1.0 mg mL^−1^), and Light irradiation (3 min at 1.0 W cm^−^^2^) groups exhibited sustained growth, suggesting the aggressive and fast‐progressing nature of fibrotic breast cancer. However, in the αPD‐1 (5 mg kg^−1^) group, the right primary tumor showed rapid growth, whereas the left tumor had its growth inhibited due to the absence of fibrosis. This is because the nonfibrotic microenvironment of the recurrent tumor allows better antibody penetration, while the fibrotic stroma of the primary tumor limits it. In contrast, the FAP‐IR1061 + Light treatment group showed the opposite result in tumor growth. When PTT was combined with αPD‐1, the primary and recurrent tumors demonstrated nearly stagnant volume, with only minor increases observed in some cases. This enhanced suppression of both the primary and recurrent tumor indicates that PTT‐induced immune activation synergizes with αPD‐1 to inhibit distant tumor growth.

As illustrated in Figure 10G, the absence of abnormal weight changes during treatment confirms the safety of PTT and ICB therapy. At the same time, as illustrated in Figure 10D,J, spleen size and volume in the FAP‐IR1061 + Laser + αPD‐1 group and αPD‐1 group decreased posttreatment, suggesting tumor‐induced splenomegaly was effectively mitigated. Simultaneously, αPD‐1 increased the infiltration of CD8^+^ CTLs and GZMB^+^ CTLs in recurrent tumors. Remarkably, the integration of PTT with αPD‐1 also resulted in greater infiltration of GZMB^+^ CTLs into recurrent tumor (Figure 10K). The findings suggest that the ECM within the TME. The results further indicated that the ECM in the TME serves as a crucial physical barrier affecting TIL penetration. Additionally, it can be inferred that PTT combined with immunotherapy can remodel the ECM to enhance immune cell infiltration and immune activation in tumor tissues. BL imaging was also used to facilitate monitoring tumor growth in the treatment (Figure 10L and Figure S13). The results of BL imaging also demonstrated the inhibitory effect of the combined therapy on recurrent tumors, as well as the effect of αPD‐1 releasing the tumor immune brake after remodeling the ECM in the TME. There was a rise in proinflammatory cytokines, including Interleukin 1β (IL‐1β) and TNF‐α, which activate T cells, along with an increase in GZMB and IFN‐γ for T cell cytotoxic effects (Figure 10M). The changes in cytokine levels in vivo, also reflected the immunomodulatory effects caused by PTT and αPD‐1. Based on the above results, only the combined therapy group successfully inhibited tumor recurrence.

## 3 Discussion

In this study, we measured FAP expression in TNBC, and identified the pivotal role of CAFs in promoting the tumor proliferation, migration, and poor prognosis. Given the high FAP levels observed, we successfully developed a multifunctional NPs by conjugating FAP with DSPE‐IR1061, enabling specific inhibition of CAFs and modulation of the ECM. Unlike the passive or direct tumor cell targeting strategies used in many of these studies [85, 86, 87, 88, 89], the nanoplatform exhibits exceptional capabilities for dynamic, noninvasive, and quantitative visualization of FAP by the effective integration of NIR‐II fluorescence imaging, PA imaging, and MR imaging. Remarkably, MRI signal enhancement correlated strongly with probes accumulation, offering a unique tool with potential for clinical translation in vivo FAP quantification. This capability supports noninvasive identification of FAP‐high tumors pretreatment, laying a strong material and imaging foundation for personalized CAFs‐targeted therapy.

Rather than directly attacking tumor cells, we focused on CAFs, one of stromal component in the TME, as emerging target for therapy. We confirmed the tumor‐promoting function of FAP^+^ CAFs in driving proliferation and migration, linked to activation of key cancer hallmark pathways, such as TGF‐β, PI3K‐AKT, MAPK, and JAK‐STAT signaling pathway and so on [13, 41]. Transcriptome sequencing and mIF revealed that CAFs deposit ECM components such as α‐SMA, FN, and collagen, collectively forming a physical barrier that reinforces tumor growth and immune suppression. FAP‐targeted PTT counteracted these effects by inducing oxidative stress [13, 41], suppressing pro‐tumor signaling pathways [13, 41], and promoting antigen presentation [38, 39]. Additionally, PTT holds therapeutic potential as a treatment option due to its ability to boost the efficacy of immunotherapy by triggering ICD [21, 22, 23, 24, 36, 37]. As FAP is also minimally expressed in tumor epithelium, laser‐triggered PTT via FAP‐IR1061 enhances ICD in tumor cells by inhibiting tumor drug resistance pathways [13, 41], ameliorates abnormal tumor vessels [42], alleviates high cellular stress [13, 41], and blocks tumor survival and invasion. Ultimately, ICD led to DC activation [21, 22, 23, 24, 36, 37], TANs repolarization [38, 39] and CTL recruitment [13, 34, 35], thereby shifting the TME from a chronically inflamed state to a potent T cell‐mediated antitumor response.

By integrating stromal‐targeted PTT with immunotherapy, we devised a synergistic strategy to reverse the immunosuppressive landscape and overcome ICB resistance. Our nanoprobes unites targeted multimodal imaging and efficient PTT in a single nanoplatform. Physical ablation of CAFs remodels the ECM barrier, amplifies ICD, and initiates a systemic immune cascade that potentiates ICIs. In summary, this work introduces an integrated and promising approach combining FAP‐targeting MR/PA imaging/NIR‐II fluorescence with PTT‐immunotherapy for TNBC, providing a compelling strategy for ECM reprogramming and enhanced immune treatment efficacy.

## 4 Experimental Section

### 4.1 Materials

The Lyso‐Tracker, DAPI solution, D‐luciferin potassium salt, crystal violet staining solution, puromycin, BeyoClick EdU Cell Proliferation Kit with AF488, RIPA buffer containing protease inhibitors (PMSF), TNUEL, Soaking and Activation Buffer for polyvinylidene difluoride (PVDF) membrane, BCA protein assay, were purchased from Beyotime Ltd. (Shanghai, China). PVDF membrane was obtained from Millipore (Darmstadt, Germany). CCK‐8 assay kit was obtained from Absin. 4% paraformaldehyde and PBS were obtained from Biosharp (Beijing, China). Bovine Serum Albumin (BSA) were purchased from Sigma‐Aldrich (Missouri, USA). Fetal bovine serum (FBS) was purchased from ExCell Bio (Suzhou, China). RPMI‐1640 (Gibco), Dulbecco’ s modified Eagle’ s medium (DMEM, Gibco), penicillin–streptomycin solution (PS, Gibco), Mito‐Tracker was sourced from Thermo Fisher Scientific Inc (Shanghai, China). The millipore filtration system was used for water purification. Anti‐FAP‐antibody was sourced from Signalway Antibody (SAB, Maryland, USA, #54991‐1). Anti‐ α ‐ SMA (80008‐1‐RR), Anti‐FN (156131‐AP), Anti‐type I Collagen (66761‐1‐Ig), Anti‐Calreticulin (CRT, 10292‐AP), Anti‐high mobility group box 1 (HMGB 1, 110829‐AP), were obtained from Proteintech (Chicago, USA). Ncm Enhanced Chemiluminescent (ECL) Ultra Reagent was obtained from NCM Biotech (Suzhou, China). Gd‐DTPA were obtained from Guerbet (Paris, France). Anti‐PD‐1(αPD‐1, Pembrolizumab) was obtained from Selleck chemicals (Houston, USA). ACK Lysing Buffer was obtained from Yuanye Bio‐Technology Co., Ltd (Shanghai, China).

### 4.2 Bioinformatics’ Analysis

The TCGA Breast Cancer dataset was retrieved from UCSC Xena (https://xena.ucsc.edu/), and the GEO Breast Cancer dataset was obtained from the UCSC Genome Browser database (http://genome.ucsc.edu).

### 4.3 Cell Culture and the Construction of the CAFs Model

CAFs activation is driven by hypoxia in the TME and various cytokines [13]. Tumor cells secrete TGF‐β, activating surrounding normal fibroblasts and convert them into CAFs [43]. In vitro studies, activated NIH 3T3 cells were harvested to establish the CAFs model for the relevant cellular experiments. 4T1 and NIH 3T3 cells were cultured in RPMI‐1640 and DMEM, respectively, each supplemented with 10% FBS and 1% PS solution, at 37°C in a 5% CO_2_ humidified environment. Then NIH3T3 cells were exposed to the CM from 4T1 cells in a culture dish or in a six‐well plate for 24 h. The activated NIH3T3 cells, identified as CAFs, were selected for the relevant experiments [90, 91, 92, 93, 94, 95].

### 4.4 Construction of Mouse Tumor Xenograft Model

The animals used in the experiment were 4 weeks‐old female BALB/c mice. All animal experiments in this study were approved by the Institutional Animal Care and Use Committee of Xinhua Hospital, affiliated to Shanghai Jiao Tong University School of Medicine (XHEC‐F‐2024‐014). Previous studies have shown that subcutaneous tumor‐bearing mice implanted with NIH3T3 cells can be used as a fibrotic breast cancer model [43, 94, 96]. However, there were no reports related to the in situ tumor model of breast adipose tissue to date. To establish a CAFs‐enriched in situ breast cancer model, 4T1 cells were mixed with CAFs at 2:1 ratio and injected the cell suspension subcutaneously into the right second mammary fat pad of each mouse [97]. A conventional 4T1 breast cancer model was also established as a control. Mice in the CAFs‐enriched in situ breast cancer model group were injected with 5 × 10^5^ 4T1 cells and 2.5 × 10^5^ CAFs, while those in the conventional 4T1 breast cancer model were injected 5 × 10^5^ 4T1 cells.

To investigate the effects of PTT‐immunotherapy on primary tumors, a mixed solution of luciferase‐expressing 4T1 cells and CAFs was implanted into the right second mammary fat pads of the mice. Further, a recurrent tumor model was established as follow: a mixed solution of 4 T1 cells and CAFs was implanted into the right second mammary fat pad of the mice, while only luciferase‐expressing 4T1 cells were implanted into the left side. Tumors typically developed approximately 3 days after cell injection. In vivo animal imaging experiments were performed at approximately 1‐week postinjection, when tumors reached a diameter of 8–12 mm. Due to considerations for animal welfare, the mice were euthanized by rapid cervical dislocation.

### 4.5 Synthesis of the FAP‐Targeting Peptide

The FAP‐targeted peptide, functionalized with a NOTA chelator, was synthesized on Rink amide resin using standard Fmoc‐based solid‐phase peptide synthesis (SPPS) on a CEM Liberty One microwave‐assisted peptide synthesizer. For MR imaging, FAP‐targeted peptide was further chelated with Mn^2^^+^ ions (1.0 mm) through coordination with a NOTA chelator. The crude product was purified by preparative high‐performance liquid chromatography (HPLC) using a C‐18 reverse‐phase column. The purified peptide was characterized by analytical HPLC and high‐resolution mass spectrometry performed on a Waters Micromass Q‐TOF spectrometer equipped with an electrospray ionization source (Figures S14 and S15).

### 4.6 Preparation of the FAP‐Targeted NIR‐II/MR Dual‐Modal Nanoprobes

For NIR fluorescent imaging, the NIR‐II fluorescent dye IR‐1061 was encapsulated inside Mal‐DSPE‐PEG_2000_ liposomes, and the liposomes were subsequently surface‐modified with the FAP‐targeting peptide. In details, IR‐1061‐loaded liposomes were fabricated using the thin‐film hydration technique. Specifically, Mal‐DSPE‐PEG_2000_ (10 mg) was dissolved in 4 mL of a chloroform/methanol mixture (3:1 v/v), followed by solvent evaporation to create a lipid film. This film was hydrated with PBS (pH 7.4) containing IR1061 (1.0 mg mL^−1^) to form uniform liposomes, resulting in the formation of IR1061 NPs. For FAP‐targeting peptide conjugation, thiol‐containing peptides were conjugated to the maleimide groups on Mal‐DSPE‐PEG_2000_ via Michael addition reaction in PBS (pH 7.4). To obtain the final FAP‐targeted nanoprobes, the product was dialyzed in ultrapure water for 24 h followed by lyophilization, which was named FAP‐IR1061 NPs. For in vitro fluorescence imaging, Cy5‐labeled FAP‐IR1061 was prepared by conjugating thiol‐containing fluorescent Cy5‐SH (1.0 mm) to the remaining maleimide groups on the nanoprobes via Michael addition in PBS (pH 7.4) for 2 h at room temperature (Table S2).

### 4.7 Characterization of the FAP‐Targeting Probes

High‐resolution images of FAP‐IR1061 NPs and IR1061 NPs were obtained via a TEM. The sample preparation process involves depositing a few drops of FAP‐IR1061 NPs and IR1061 NPs solutions onto a holey carbon‐coated grid, followed by drying at room temperature. TEM analysis was performed using a JEM‐2100 electron microscope (manufactured by JEOL, Japan). The hydrodynamic diameter and colloidal stability of FAP‐IR1061 NPs and IR1061 NPs were evaluated using DLS immediately after preparation and after 1 day, 3 days, and 7 days of incubation in three different media: PBS, RPMI‐1640 cell culture medium, and 0.9% saline (Figure 3A and Figure S3A,B,C). The zeta potential of the material was measured using a Nano‐Zeta Instrument (Zetasizer, Malvern). Both the fluorescence and absorption spectra of the probes were measured using a Varian Cary 100 Conc UV–Vis spectrometer and a Varian Cary Eclipse spectrometer (Edinburgh Instruments Ltd). Solutions of FAP‐IR1061 NPs and IR1061 NPs were prepared in PBS, and their UV–Vis absorption spectra and fluorescence spectra were detected at 25°C.

### 4.8 In Vitro NIR‐II Imaging, PA Imaging and MRI

NIR‐II fluorescence imaging system (MARS) captured the NIR‐II images and intensities of FAP‐IR1061 and IR‐1061 NPs in PBS solution under 980 nm illumination (100 mW cm^−^^2^). ImageJ software was used to generate pseudocolor images. PA images and intensities of FAP‐IR1061 NP at 980 nm were acquired using a PA microscopy system (Vevo LAZR, VisualSonics, a tunable laser source with a wavelength range of 680–970 nm). For ex vivo T1‐weighted relaxometry measurements, samples were diluted in water with concentrations of 0.025, 0.05, 0.1, 0.25, 0.5, and 1.0 mm before being measured with a 9.4 T Bruker BioSpec. The instrument parameters were set as follows: field of view (FOV) = 3.5 cm × 3.0 cm; slice thickness = 1 mm; slice gap = 1 mm; repetition time (TR) = 500 ms; echo time (TE) = 4.85 ms; flip angle = 180°, number of averages = 2; image size = 200 × 172. The longitudinal relaxation rate (r1) was calculated via linear regression of 1/T1 (sec^−1^) against the probes concentration.

### 4.9 In Vitro and Vivo Photothermal Performance

Photothermal images of FAP‐IR1061 NPs were captured with an infrared thermal imaging camera. FAP‐IR1061 NPs (1.0 mg mL^−1^) were diluted in PBS to prepare solutions of different concentrations. Subsequently, these FAP‐IR1061 NPs (6.25–200 µg mL^−1^) underwent irradiation with a different NIR laser (0.8–1.6 W cm^−2^). Temperature changes of the FAP‐IR1061 NPs were monitored every 10 s on the infrared thermal imaging camera. The PCE (*η*) was determined based on the method reported by Roper et al. [98]. The efficiency was calculated using the following equation:

(1) $$\eta =\frac{hS\left(T_{\max}-T_{env}\right)-Q_{0}}{P\left(1-10^{-A_{\lambda }}\right)}$$

where *T*_max_ and *T*_env_ are the maximum and environmental temperatures, respectively. *Q*_0_ is the nonspecific power absorbed by the PBS, *P* is the laser power, *A*_λ_ is the absorption value of FAP‐IR1061 NPs at 980 nm. *h* is the heat transfer coefficient, and *S* is the surface area irradiated during PTT. All of these terms except the *hS* can be obtained experimentally. The *hS* value was determined by analyzing the cooling profile according to the reported method using another equation [99]:

(2) $$hS=\frac{mC_{p}}{\tau }$$

Here, *m* and *C*_p_ are the mass and specific heat capacity of water, respectively. *τ* is the time constant derived from the linear relationship between cooling time and −ln(*θ*) (Figure S4B) and where *θ* = Δ*T*/Δ*T*_max_.

BALB/c mice bearing CAFs‐enriched breast cancer model were divided into 2 groups (*n* = 5), and injected with FAP‐IR1061 NPs (100 µL, 100 µg mL^−1^) and PBS (100 µL) via the tail vein, respectively. Further, the tumor of these mice was irradiated by NIR laser after 6 h. Temperature changes at the tumor sites of mice treated with FAP‐IR1061 NPs were monitored following the same protocol as above.

### 4.10 In Vivo Fluorescence Imaging, PA Imaging, and MRI

BALB/c mice in CAFs‐enriched breast cancer model and conventional 4T1 breast cancer model were divided into 4 group (*n* = 3). They were injected with either FAP‐IR1061 NPs or IR1061 NPs (100 µL, 100 µg mL^−1^) via the tail vein, and then scanned for NIR‐II fluorescence imaging at 2, 4, 6, 8, 10, 12, and 24 h using MARS with emission light filtered through an LP 980 filter. At the 8 h, the mice were euthanized and their major organs were dissected for NIRF analysis using to MARS. In addition, PA imaging was performed at 2, 4, 6, 8, 10, and 24 h using a PA microscopy system (Vevo LAZR, VisualSonics, a tunable laser source with a wavelength range of 680–970 nm).

Furthermore, mice in the CAFs‐enriched breast cancer model were intravenously injected with FAP‐IR1061 NPs (100 µL, 1.0 mm). MR imaging was conducted before injection and at 1, 3, 5, and 8 h postinjection. T1‐weighted rapid acquisition with relaxation enhancement (RARE) axial images were obtained using a 9.4 T Bruker BioSpec system with the instrument parameters sets the same as those for the aforementioned ex vivo experiments. The longitudinal relaxation rate (r1) was obtained by linear regression of 1/T1 (sec^−1^) versus the concentration of the probes.

### 4.11 In Vivo Luminescence Imaging of Primary Breast Cancer and Recurrent Breast Cancer during Treatment

BALB/c mice bearing primary CAFs‐enriched in situ breast cancer model and recurrent breast cancer model were intraperitoneally injected with D‐luciferin potassium salt (10 min prior to imaging). Bioluminescence were performed using the IVIS system.

### 4.12 Cellular Uptake and Colocalization Analysis

CAFs and 4T1 cells were exposed to 5.0 µm FAP‐IR1061(Cy5 labeled) for different time points (1, 2, 3, and 4 h) to identify the optimal incubation time for probes intracellular internalization. To further confirm FAP expression and the fluorescence intensity of FAP‐IR1061(Cy5 labeled) in the cells, CAFs and 4T1 cells were treated with FAP‐IR1061(Cy5 labeled) for 2 h, followed by incubation with 50 nm Lyso‐Tracker for 30 min and then with DAPI for 10 min. After washing the cells three times with PBS, images were recorded using a confocal laser scanning microscope (CLSM, TCS SP5, Leica, Germany). Subsequently, the fluorescence intensity and organelle co‐localization coefficient were analyzed using ImageJ software.

### 4.13 Immunofluorescence Staining

CAFs, NIH 3T3 cells, and 4T1 cells were cultured in 6‐well plates for a duration of 24 h and then fixed with 4% paraformaldehyde. The fixed cells incubated with 5% BSA 1 h, then with diluted with diluted FAP‐specific primary antibodies overnight at 4°C. For mIF, CAFs as well as NIH 3T3 cells incubated overnight at 4°C with diluted primary antibodies specific for α‐SMA, FN, and type I collagen. Following this, the cells was washed with PBS and exposed to a fluorescently labeled secondary antibody for 1 h to finalize the staining procedure. The cells were then washed twice with PBS and mounted in a DAPI‐containing mounting medium. Fluorescence images were captured using an Olympus X81 epifluorescence microscope.

Additionally, CAFs and 4T1 cells were separately cultured in a confocal petri dish and incubated overnight to reach 70–80% confluency. The cells were subsequently exposed to a FAP‐IR1061 NPs at concentrations ranging from 50 to 200 µg mL^−1^ for a duration of 4 h, respectively. Then, the cells were either subjected to NIR laser or kept in the dark as the control. Living and dead cells were distinguished using the fluorescent probes calcein‐AM/PI after 24 h. Fluorescence Images were captured using an Olympus X81 epifluorescence microscope.

### 4.14 EdU Assay Analysis

4T1 cells were incubated in 24‐well plates for a duration of 24 h. The original culture CM was removed, followed by the addition of RPMI‐1640 containing CM from either CAFs or CAFs post‐PTT. The BeyoClick EdU‐488 cell proliferation detection kit was used to evaluate the influence of different media on 4T1 cell proliferation. Images were captured using an inverted fluorescence microscope, and cell counting as well as calculations of the proportion of EdU‐positive cells were performed using ImageJ software.

### 4.15 Scratch Wound Healing Assay

4T1 cells were cultured in 6‐well plates for a duration of 24 h. When the cells reached confluence, a scratch was made in each well, and the cells were rinsed with PBS. Subsequently, RPMI‐1640 medium containing CM from either CAFs or CAFs post‐PTT was added, which either contained 10% FBS or no FBS. The scratch healing process was monitored and recorded after 24 h. Ultimately, ImageJ software was used to measure the scratch area.

### 4.16 Migration Assay

4T1 cells were cultured in the upper chamber of Transwell inserts, which contained no FBS, while the lower chamber contained RPMI‐1640 supplemented with CM from either CAFs or CAFs post‐PTT, with 10% FBS added. After an additional 24 h, the cells were fixed, stained, and observed with an inverted fluorescence microscope. ImageJ was utilized for cell counting.

### 4.17 In Vitro Cytotoxicity Assays

To assess the cytotoxicity of FAP‐IR1061 NPs, 4T1, and CAFs cells were cultured in 96‐well plates and exposed to varying concentrations of FAP‐IR1061 NPs (0–200 µg mL^−1^) for 4 h. Subsequently, the cells were either treated with irradiation or kept in the dark for 3 min each well. In addition, CAFs and 4T1 cells were incubated with FAP‐IR1061 NPs (100 µg mL^−1^) for 4 h, while the control group was maintained in DMEM and RPMI‐1640, respectively. Then, the cells underwent a NIR laser for 3 min per well at different power densities. To assess relative cell viability, 100 µL of enhanced CCK‐8 solution was added to each well. The Spectramax Microwell Plate Reader from Thermo Fisher Scientific Absorbance was utilized to measure the absorbance at 450 nm, and relative cell viability was then calculated.

### 4.18 Western Blot

CAFs, NIH3T3 cells, and 4T1cells were cultured in 6‐well plates for 24 h. Subsequently, RIPA buffer supplemented with PMSF was added to the wells for cell lysis and scraping. The extracted protein samples (10 µg) were separated and transferred onto a PVDF membrane using a transfer buffer at a current of 400 mA for 35 min, followed by overnight incubation at 4°C with diluted anti‐FAP or anti‐α‐SMA antibodies. Finally, the membrane was incubated with secondary antibody for 1 h to complete the experimental procedure. Protein bands were visualized using the BeyoECL Star kit in conjunction with the GelDocXR system (Bio‐Rad, USA). For the probes‐treated group, CAFs and 4T1 cells were exposed to FAP‐IR1061 NPs at concentrations of 0 or 100 µg mL^−1^ for 4 h, followed by either NIR laser irradiation or dark incubation for 3 min. The same steps described above were then carried out. Finally, the expression levels of FAP, α‐SMA, CRT, and HMGB 1 were quantified using ImageJ.

### 4.19 Bulk RNA Sequencing

CAFs and NIH3T3 cells were collected, and total RNA was extracted for transcriptome sequencing data. CAFs were incubated with FAP‐IR1061 NPs (100 µg mL^−1^) for 4 h. Following this, the cells were treated with NIR laser irradiation or dark incubation for 3 min per well. After 24 h, the cells were collected for RNA sequencing. After 24 h, the cells were collected for RNA‐seq. For 4T1 cells, total RNA was extracted from two groups, including one group maintained in RPMI‐1640 medium and the other incubated with RPMI‐1640 medium containing CM from CAFs. Following the euthanasia of the mice, tumors were excised and subsequently conserved in liquid nitrogen. RNA‐seq was completed by Oebiotech Company (Shanghai, China). The sequencing data was analyzed and visualized on an available and online platform at www.bioinformatics.com.cn. Genes with an adjusted *p*‐value < 0.05 and fold change >2.0 were employed to determine statistical significance.

### 4.20 Histological Analysis, Flow Cytometry Analysis and CyTOF

Following the euthanasia of the mice, tumors and major organs from all groups (*n* = 6) were excised. These tissues were either preserved in tissue storage solution or fixed in 4% paraformaldehyde. The tumor tissue was taken out of the storage solution, and CyTOF was performed by Puluoting Health Technology (Zhejiang, China). After that, the spleen was extracted from the storage solution. Red blood cells were subsequently removed using ACK lysis buffer, and the remaining tissues were digested with collagenase IV at 37°C for 1 h and filtered through a 70 µm strainer to obtain a single‐cell suspension. The isolated single cells were then resuspended in staining buffer and stained with fluorescence‐conjugated antibodies to identify specific cell populations, including CTLs (CD45^+^CD3^+^CD4^−^CD8^+^), CD4^+^ T cells (CD45^+^CD3^+^CD4^+^CD8^−^), regulatory T cells (Tregs, CD45^+^CD3^+^CD4^+^CD25^+^Foxp3^+^), and MDSCs (CD45^+^CD3^−^CD11b^+^). Data were collected using CytExpert 2.0 software and subsequently analyzed with FlowJo software (version 10.0.7r2).

Tissues were embedded in paraffin, and sections were prepared for H&E and immunofluorescence staining. Primary antibodies targeting FAP, α‐SMA, FN, type I collagen, CRT, HMGB 1, Ki67, GZMB^+^CD8^+^ CTLs, and TUNEL were utilized. These were followed by staining with an Alexa Fluor 488‐labeled anti‐mouse secondary antibody and DAPI. Blood samples were also collected from the orbital venous sinus of mice across all groups after treatment. The samples were stored at room temperature for 2 h and then centrifuged at 3000× *g*, 4°C for 10 min. The supernatant was collected, and ELISA assays were performed using commercial kits following the manufacturer's instructions.

To evaluate the in vivo retention of the probes, tumor‐bearing mice and healthy mice were intravenously injected with FAP‐IR1061 NPs (100 µL, 1.0 mm) via the tail vein. For tumor‐bearing mice, two types of tumor‐bearing mice were euthanized at 24 h postinjection, and major organs, tumors, and blood samples were collected. For healthy mice, a time‐course study was conducted. Blood samples were collected at preinjection and at 1, 2, 4, 6, 8, and 24 h postinjection. Major organs (heart, liver, spleen, lungs, and kidneys) were harvested at 4, 6, 8, and 24 h postinjection. Urine samples were collected at preinjection and at 4, 6, 8, and 24 h postinjection, while fecal samples were collected at preinjection and at 1, 4, 6, 8, and 24 h postinjection. All samples underwent microwave digestion, followed by quantitative analysis of Mn concentrations using inductively coupled plasma mass spectrometry (ICP‐MS) (Table S3).

An in vitro assessment of serum biochemistry and hemolytic effects of FAP‐IR1061 NPs was conducted to evaluate its biosafety. In hemolysis assay, FAP‐IR1061 NPs was prepared at the injection concentration and then diluted to several concentrations. For controls, physiological saline served as negative control, and ddH_2_O served as positive.

### 4.21 Statistical Analysis

Experimental data were presented as the mean ± standard deviation (SD), with error bars denoting the SD. Intergroup differences were evaluated using a two‐tailed Student's *t*‐test and two‐way analysis of variance (ANOVA). Significance levels were indicated by asterisks: *p < 0.05, **p < 0.01, ***p < 0.001, and ****p < 0.0001.

## Author Contributions

**Ling Zhan**: validation, investigation, visualization, writing – original draft. **Yanhong Chen**: validation, visualization. **Jingjing Hu**: methodology, investigation. **Chunting Wang**: methodology, investigation. **Huanhuan Liu**: conceptualization, methodology, investigation, formal analysis, writing – review and editing, funding acquisition. **Defan Yao**: conceptualization, methodology, investigation, formal analysis, writing – review and editing, funding acquisition. **Dengbin Wang**: supervision, writing – review and editing, project administration, funding acquisition. All authors read and approved the final manuscript.

## Conflicts of Interest

The authors declare no conflicts of interest.

## Supporting information




**Supporting File**: adma74089‐sup‐0001‐SuppMat.docx.

## Data Availability

The data that support the findings of this study are available on request due to privacy and ethical restrictions.
