# Supplementary material for: Reprogramming the Immunosuppressive Microenvironment in Triple‐Negative Breast Cancer via FAP‐Targeted Nanoprobes for Multimodal Imaging and Photothermal Therapy
Source: Adv Mater. 2026 Jul 15;38(47):e74089. doi: 10.1002/adma.74089 (PMC13496071; doi:10.1002/adma.74089)
Supplement: Supplementary file 1 [file ADMA-38-e74089-s001.docx]

**Supporting Information**

**Reprogramming the Immunosuppressive Microenvironment in Triple-Negative Breast Cancer via FAP-Targeted Nanoprobes for Multimodal Imaging and Photothermal Therapy**

*Ling Zhan^1#^, Yanhong Chen^1#^, Jingjing Hu^1,2^, Chunting Wang^1^, Huanhuan Liu^1*^, Defan Yao^1*^, Dengbin Wang^1,2*^*

Dr. L. Zhan, Y. Chen, J. Hu, C. Wang, H. Liu, D. Yao, D. Wang

Department of Radiology, Xinhua Hospital, Shanghai Jiao Tong University School of Medicine, 200092, Shanghai, China.

Dr. J. Hu, Prof. Dr. D. Wang

Department of Radiology, Xinhua Hospital, Shanghai Jiao Tong University School of Medicine, 200092, Shanghai, China.

Faculty of Medical Imaging Technology, College of Health Science and Technology, Shanghai Jiao Tong University School of Medicine, 200092, Shanghai, China.

E-mail: [wangdengbin@xinhuamed.com.cn (D. Wang);](mailto:wangdengbin@xinhuamed.com.cn;) yaodefan@xinhuamed.com.cn (D. Yao), liuhuanhuan@xinhuamed.com.cn (H. Liu).

Ling Zhan and Yanhong Chen contributed to this work equally.

Keywords: triple-negative breast cancer, cancer-associated fibroblasts, photothermal therapy, immunotherapy

**Materials**

TBST, 0.1% Triton X-100, Calcein-AM/PI live/dead viability/cytotoxicity kit, Goat anti-mouse (A0216) and anti-rabbit IgG-HRP antibody (A208) , and Cy3-labeled Goat anti-mouse IgG-HRP antibody (A0507) were purchased from Beyotime Ltd (Shanghai, China). 10 % sodium dodecyl sulfate−polyacrylamide gel electrophoresis (SDS-PAGE) and two-color pre-dyed marker were purchased from Vazyme (Nanjing, China). LIVE/DEAD fixable violet dead cell staining kit were sourced from Thermo Fisher Scientific Inc (Shanghai, China). Isoflurane was purchased from RWD Life Science (Shenzhen, China). Transwell chambers were obtained from Corning (New York, USA). Spot illumination (638 nm, 0.68 W) was purchased from Beijing RaySource Technology Co., Ltd. The wide-field illumination was used for light exposure on culture plates during cell experiments, while spot illumination device was used for light exposure in other experiments. Mouse IL-6 ELISA kit, Mouse IL-2 ELISA kit, Mouse Interleukin 1β ELISA kit, Mouse TNF α ELISA kit, Mouse Interferon γ ELISA kit, and Mouse GZMB ELISA kit was obtained from Uscnk (Wuhan, China).

**Bioinformatics’ analysis**

A comprehensive pan-cancer bioinformatics analysis was performed to evaluate FAP expression in tumor tissues across different cancer types. Subsequently, the expression levels of FAP in tumor tissues and adjacent normal tissues from TNBC patients were compared to clarify FAP’s differential expression. Additionally, the correlation between FAP expression and prognosis was also evaluated in TNBC patients. Using single-cell sequencing data of CAFs from the GEO database, cellular fractionation and FAP visualization were performed to determine the distribution of FAP-expressing cells. Gene Ontology (GO) functional enrichment analysis was performed to explore the association between the expression of FAP-related key hub genes and epithelial-mesenchymal transition (EMT) (e.g., ECM regulation). CellChat analysis was performed to elucidate the interaction patterns between fibroblasts and other cell types.

**Construction of mouse tumor xenograft model**

During MR imaging, the tumor in situ is too close to the heart, which will cause artifacts due to respiratory motion. Therefore, subcutaneous tumor models were used for MRI experiments in mice.

**Statistical analysis**

Experimental data were presented as the mean ± standard deviation (SD), with error bars denoting the SD. Intergroup differences were evaluated using a two-tailed Student's t-test and two-way analysis of variance (ANOVA). Significance levels were indicated by asterisks: **p* < 0.05, ***p* < 0.01, ****p* < 0.001, and *****p* < 0.0001.

**Supplementary Figures**

**Figure S1. Cellular fractionation and FAP visualization from single-cell data from GEO CAFs.**

**Figure S2.** The Gene Ontology (GO) function analysis histogram of CAFs.

**Figure S3. Characterization of FAP-IR1061 NPs and IR 1061 NPs. A)** Representative TEM images (Scale bar: 200 μm) and DLS profiles of IR-1061 NPs. **B)** DLS analysis of FAP-IR1061 NPs incubated for 1 day, 3 days and 7 days in PBS, RPMI-1640 media and 0.9 % saline, respectively. **C)** DLS analysis of IR1061 NPs incubated for 1 day, 3 days and 7 days in PBS, RPMI-1640 media and 0.9 % saline, respectively. **D-E)** Absorption (λab: 700 -1300 nm) **(D)**, and fluorescence spectra (λem: 1000 -1200 nm, λex: 980 nm) **(E)** of IR-1061 NPs in PBS. **F)** Average surface potential of FAP-IR1061 NPs and IR-1061 NPs. **G)** NIR-II ﬂuorescence images and pseudo-color images of the FAP-IR1061 NPs and IR-1061 NPs (0.00625 −1.0 mg/mL) in PBS solution under 980 nm illumination (1.0 W/cm^2^). **H)** The MR images and pseudo-color of FAP-IR1061 NPs at different concentrations (0.0125 − 1.0 mM). **I)** Quantitative curve of fluorescence signals for FAP-IR1061 NPs and IR-1061 NPs (0.00625 −1.0 mg/mL) in PBS solution under 980 nm illumination (1.0 W/cm^2^). **J)** PA images at 980 nm laser (1.0 W/cm^2^) of FAP-IR1061 NPs (0.05, 0.1, 0.5, 1.0 mg/mL). **K)** The fitting curves of PA intensity of FAP-IR1061 NPs at 980 nm against concentrations (1.0 W/cm^2^). **L)** PA spectrum of FAP-IR1061 NPs (1.0 W/cm^2^).

**Figure S4. Photothermal stability and temperature response of FAP-IR1061 NPs.** **A)** Temperature changing curves of the FAP-IR1061 NPs solution and ICG in the presence of 980 nm laser irradiation (1.0 W/cm^2^) at 50 μg/mL. **B)** The temperature rise and decay curve of FAP-IR1061 NPs under 980 nm laser irradiation at 50 μg/mL (1.0 W/cm²). **C)** Temperature curves of the FAP-IR1061 NPs (50 μg/mL) solution during three consecutive cycles “on/off” irrdiation with 980 nm laser (1.0 W/cm^2^).

**Figure S5.** **A)** NIR-II fluorescent distribution of CAFs-enriched 4T1 tumors and 4T1 tumors at different time points after intravenous injection of IR1061 NPs (100 μL, 1.0 mg/mL). **B)** *In vivo* NIR-II fluorescence quantification of tumor tissues for IR1061 NPs (100 μL, 1.0 mg/mL), n = 5, data present as mean ± SD (**p* < 0.05). **C)** NIR-II fluorescence quantification of tumor tissues and major organs from CAFs-enriched 4T1 tumors and 4T1 tumors at 8 h after injection of IR1061 NPs (100 μL, 1.0 mg/mL). **D)** Hematoxylin and eosin (H&E) staining images of major organs after injecting with IR1061 NPs (100 μL, 1.0 mg/mL) or PBS, Scale bar: 100 μm. **E)** Quantitative analysis of NIR-II fluorescence intensity of IR1061 NPs (100 μL, 1.0 mg/mL) in tumor tissues and major organs, n = 5, data present as mean ± SD (**p* < 0.05).

**Figure S6.** **A)** *In vivo* PA imaging of CAFs-enriched 4T1 tumors and 4T1 tumors at different times after intravenous injection of FAP-IR1061 NPs (100 μL, 1.0 mg/mL). Scale bar is 2 mm. **B)** The fitting curves of PA intensity for FAP-IR1061 NPs (100 μL, 1.0 mg/mL) at 980 nm in CAFs-enriched 4T1 tumors and 4T1 solid tumor model. **C)** Hemolysis experiment of FAP-IR1061 NPs (100 μL, 1.0 mg/mL). **D)** Liver function makers (ALT, AST, ALP) and kidney function makers (UREA, CREA) of mice in each group after adminstrating FAP-IR1061 NPs (100 μL, 1.0 mg/mL). **E)** Distribution of Mn in major organs and tumor of two types of tumor-bearing mice at 24 h post-injection of FAP-IR1061 NPs (100 μL, 1.0 mM). **F)** Time course of Mn concentrations in the blood of healthy mice at pre-injection and at 1, 2, 4, 6, 8, and 24 h after intravenous injection of FAP-IR1061 NPs (100 µL, 1.0 mM). **G)** Time-dependent bio-distribution of Mn in major organs (heart, liver, spleen, lungs, and kidneys) of healthy mice at 4, 6, 8, and 24 h after intravenous injection of FAP-IR1061 NPs (100 µL, 1.0 mM). **H)** Time course of Mn concentrations in the urine of healthy mice at pre-injection and at 4, 6, 8, and 24 h after intravenous injection of FAP-IR1061 NPs (100 µL, 1.0 mM). **I)** Time course of Mn concentrations in the feces of healthy mice at pre-injection and at 1, 4, 6, 8, and 24 h after intravenous injection of FAP-IR1061 NPs (100 µL, 1.0 mM).

**Figure S7. A-B)** Gene Set Enrichment Analysis (GSEA) of CAFs pre-PTT and post-PTT, NES > 1 **(A)** indicates upregulation, and NES < -1 **(B)** indicates downregulation. *p* ＜0.05.

**Figure S8. A)** Representative H&E staining of major organs in all groups. **B)** Spleen weights of mice in all groups, n = 6, data present as mean ± SD (***p* < 0.01)**. C-E)** Quantitative analysis of the mean immunofluorescence intensity (MFI) for FAP **(C)**, CRT **(D)**, and HMGB 1 **(E)** in tumors after different treatments, n = 3, data are presented as mean ± SD (**p* < 0.05, ***p* < 0.01, ****p* < 0.001, *****p* < 0.0001)**. F)** Immunofluorescence images of TUNEL in tumors after different treatments, TUNEL: red channel, λex = 561 nm, λem = 600 - 650 nm; DAPI: blue channel, λex = 405 nm, λem = 450 - 500 nm. Scale bar: 100 μm. **G-J)** Quantitative analysis of the MFI for Ki-67 **(G),** TUNEL **(H)**, CD8^+^ CTLs **(I)**, and GZMB^+^ CTLs **(J)** in tumors after different treatments, n = 3, data present as mean ± SD (**p* < 0.05, ***p* < 0.01, ****p* < 0.001, and *****p* < 0.0001)**.**

**Figure S9.** **A)** Kyoto Encyclopedia of Genes and Genomes (KEGG) pathway analysis of the FAP-IR1061 + Light group and the PBS group ( *p*＜0.05). **B)** Gene Set Enrichment Analysis (GSEA) of the FAP-IR1061 + Light group and the PBS group (NES＞1, *p*＜0.05). **C)** KEGG pathway analysis of the αPD-1 group and the PBS group (*p*＜0.05). **D)** GSEA of the αPD-1 group and the PBS group (NES＞1, *p*＜0.05).

**Figure S10.** **A)** Kyoto Encyclopedia of Genes and Genomes (KEGG) pathway analysis of the FAP-IR1061 + Light + αPD-1group and the PBS group ( *p*＜0.05). **B)** Gene Set Enrichment Analysis (GSEA) of the FAP-IR1061 + Light + αPD-1group and the PBS group (NES＜-1, *p*＜0.05). **C)** GSEA of the FAP-IR1061 + Light + αPD-1group and the FAP-IR1061 + Light group (NES＜-1, *p*＜0.05). **D)** GSEA of the FAP-IR1061 + Light + αPD-1group and the αPD-1 group and the PBS group (NES＜-1, *p*＜0.05).

**Figure S11.** **A)** Heatmap to visualize total immune cells in the TME. **B)** T-distributed stochastic neighbor embedding (TSNE) graphs colored by 34 clusters of total immune cells. **C)** TSNE graphs of markers CD38 and CD86 for total cells. **D)** The frequency of conventional dendritic cells (cDCs), n=3, data present as mean ± SD (*p < 0.05). **E)** Heatmap to visualize T cells in the TME. **F)** The frequency of CD8 effector T cells (CD8Teff), n=3, data present as mean ± SD (*p < 0.05). **G)** Heatmap to visualize neutrophils in the TME. **H)** TSNE graphs of markers CD38, CD86,CD11b, Ly_6C for neutrophils. **I)** TSNE graphs colored by 19 clusters of neutrophils. **J)** The frequency of different neutrophils. n = 6, data present as mean ± SD (**p* < 0.05) .

**Figure S12. A)** Gene Set Enrichment Analysis (GSEA) of the FAP-IR1061 + Light group and the PBS group (NES＜-1, *p*＜0.05). **B)** GSEA of theαPD-1group and the PBS group (NES＜-1, *p*＜0.05). **C)** GSEA of the FAP-IR1061 + Light + αPD-1group and the PBS group (NES＞1, *p*＜0.05). **D)** GSEA of the FAP-IR1061 + Light + αPD-1group and the FAP-IR1061 + Light group (NES＞1, *p*＜0.05). **E)** GSEA of the FAP-IR1061 + Light + αPD-1group and the αPD-1 group (NES＞1, *p*＜0.05).

**Figure S13.** Quantified tumor bioluminescence (BL) intensities of the treatment response in BALB/c mice, n = 6, data present as mean ± SD (*****p* < 0.0001).

**Figure S14.** High-performance liquid chromatography (HPLC) analysis of FAP-NOTA-Mn.

**Figure S15.** Mass spectrometry analysis of FAP-targeting peptide, FAP-NOTA-Mn.

**Supplementary Tables:**

**Supplementary Table 1. ICP-MS Detection of Pharmacokinetics**

| **Parameter** | **Value** |
| --- | --- |
| **t₁/₂ (elimination)** | **~ 0.5 – 1 h** |
| **AUC₀₋₂₄** | **~ 3095 ng·h/mL** |
| **Clearance** | **~ 1.78 mL/h (89 mL/h/kg)** |

**Supplementary table 2. Distinct Excitation and Emission Profiles of IR1061 and Cy5**

| Fluorophore | Ex/Em (nm) | Imaging Window |
| --- | --- | --- |
| IR1061 | ~1060 nm excitation/~1000-1700 nm emission | NIR-II |
| Cy5 | ~650 nm excitation/~670 nm emission | NIR-I |

**Supplementary Table 3. ICP-MS Detection of Mn in Biodistribution, Pharmacokinetics, and Excretion**

| **Subject** | **Sample** | **Time Points** | **Purpose of Analysis** |
| --- | --- | --- | --- |
| Healthy mice | Major organs | Post-injection: 4 h, 6 h, 8 h, 24 h | Quantitatively evaluate organ distribution and accumulation |
|  | Blood | Pre-injection;  Post-injection: 1 h, 2 h, 4 h, 6 h, 8 h, 24 h | Pharmacokinetic parameters (t₁/₂, AUC, clearance) |
|  | Urine | Pre-injection;  Post-injection: 4 h, 6 h, 8 h, 24 h | Excretion pathway & clearance efficiency |
|  | Feces | Pre-injection;  Post-injection: 1 h, 4 h, 6 h, 8 h, 24 h |  |
| Two types of tumor-bearing mice | Major organs | 24 h post-injection | Quantitatively evaluate organ distribution and accumulation |
|  | Tumors |  |  |
